# Alopecia Areata: Pathogenesis, Diagnosis, and Therapies

**DOI:** 10.1002/mco2.70182

**Published:** 2025-04-21

**Authors:** Tianyou Ma, Tingrui Zhang, Fengze Miao, Jun Liu, Quangang Zhu, Zhongjian Chen, Zongguang Tai, Zhigao He

**Affiliations:** ^1^ Department of Pharmacy Longhua Hospital of Shanghai University of Traditional Chinese Medicine Shanghai China; ^2^ Shanghai Skin Disease Hospital School of Medicine Tongji University Shanghai China; ^3^ Shanghai Engineering Research Center of External Chinese Medicine Shanghai China

**Keywords:** alopecia areata, autoimmunity, diagnosis, pathogenesis, targeted therapy

## Abstract

Alopecia areata (AA) is a complex, chronic inflammatory skin disorder characterized by unpredictable, nonscarring hair loss, affecting millions worldwide. Its pathogenesis remains poorly understood, driven by intricate interactions among immune dysregulation, genetic predisposition, and environmental triggers. Despite significant advances in identifying these contributing factors, substantial gaps persist in our understanding of the full spectrum of AA's molecular mechanisms and in the development of effective therapeutic approaches. This review aims to comprehensively explore the immunological, genetic, epigenetic, and environmental factors underlying AA, with a focus on immune‐mediated mechanisms. We also evaluate diagnostic approaches and recent advancements in assessing disease severity. Furthermore, the review discusses evolving therapeutic options, including traditional therapies, biologics, small‐molecule agents, and emerging treatments. The academic value of this work lies in its synthesis of current knowledge on the multifaceted nature of AA, providing insights for future research and clinical practice. By elucidating the interconnected factors underlying AA, this review seeks to advance both understanding and management of this prevalent, clinically challenging disorder.

## Graphical abstract

This review probed into the immune factors in the pathogenesis of AA, focusing on the small molecule drugs and biologics for the treatment of AA, and anticipated the research direction in terms of the pathogenesis and targeted therapy of AA. It aims to emphasize the necessity for further exploration of its immunological pathogenesis and therapeutic strategies.

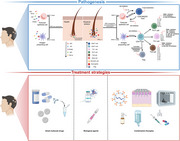

## 1 Introduction

Alopecia areata (AA), an autoimmune condition, is characterized by patchy or total nonscarring alopecia on the body and scalp [1]. Clinically, AA manifests in diverse forms, including patchy alopecia, ophiasis, sisaipho, diffuse alopecia, perinevoid alopecia, Marie Antoinette and Thomas More syndrome, alopecia areata incognita (AAI), alopecia totalis (AT), and alopecia universalis (AU). Epidemiological data indicate that AA affects individuals across all age groups, genders, and ethnicities [2]. Prevalence rates vary geographically and by age, with higher incidence in children than adults and comparable rates between genders [3, 4]. Currently, AA ranks as the second most common hair loss disorder globally, affecting approximately 2% of the population‐roughly 147 million individuals‐with projections suggesting continued growth [2]. Extensive clinical research underscores AA's profound impact on psychological health, including anxiety, insomnia, and diminished self‐esteem, particularly in severe cases [5]. Psychological distress affects nearly 60% of AA patients, with anxiety‐related disorders more prevalent among women [6, 7]. Additionally, AA patients face an elevated risk of comorbidities such as autoimmune disorders, hypertension, and hyperlipidemia [8, 9, 10]. Despite ongoing research, the precise etiology of AA remains elusive, complicating treatment development. Current therapies provide symptomatic relief but lack curative efficacy, contributing to financial strain and reduced quality of life for patients [11, 12].

Undoubtedly, immunological dysregulation plays a central role in AA's pathophysiology. Previous studies demonstrate that AA disrupts the hair growth cycle, forcing affected follicles into premature telogen and catagen phases while arresting anagen progression at stage III [13]. This process is mediated by immune cells (e.g., T cells, natural killer [NK] cells, dendritic cells [DCs], and macrophages) and driven by cytokines such as interferon‐γ (IFN‐γ) and tumor necrosis factor‐α (TNF‐α) [14]. The resulting cytokine cascade, often involving the Janus kinase (JAK)–signal transducer and activator of transcription (STAT) pathway, recruits additional immune cells, sustaining perifollicular inflammation. The dominant immunologic theory posits that AA arises from ectopic expression of hair follicle autoantigens or localized inflammatory damage triggered by factors like trauma, infection, or stress. This disrupts the hair follicle immune privilege (IP), upregulating major histocompatibility complex (MHC) class I and II antigens expression and culminating in autoimmune attack [15, 16, 17]. AA's etiology is further complicated by genetic susceptibility, epigenetic modifications, hair cycle dysregulation, and environmental influences. Diagnosis primarily relies on clinical presentation, supplemented by auxiliary tests such as hair pull tests, dermoscopy, and histopathological examination. Conventional therapies include topical contact sensitizers such as diphenylcyclopropenone (DPCP) and squaric acid dibutylester (SADBE), along with systemic or topical glucocorticosteroids. Systemic immunosuppressants such as methotrexate, azathioprine, and cyclosporine (CsA) are also commonly prescribed [18, 19, 20, 21, 22, 23, 24]. Investigational approaches include cryotherapy, methylaminolevulinic acid‐based photodynamic therapy, topical calcineurin inhibitors (CNIs), topical prostaglandin analogs (e.g., latanoprost or bimatoprost), pulsed infrared diode laser therapy, and antihistamines [25, 26]. However, many conventional treatments exhibit limited efficacy, adverse effects, poor compliance, and high relapse rates postdiscontinuation, necessitating rigorous clinical monitoring [19, 27]. Emerging therapies, notably biologics and small‐molecule drugs like JAK and phosphodiesterase 4 (PDE4) inhibitors, show promise as targeted interventions for AA [28, 29]. Compared with traditional therapies, these agents act rapidly, demonstrate superior efficacy, and enable precise modulation of disease pathways. Thus, biologics and small molecules represent a paradigm shift in AA management, with targeted therapies poised to redefine treatment standards.

This review addresses critical gaps in the academic and clinical understanding of AA by elucidating its pathogenesis, diagnostic methods, and therapeutic strategies. We aim to provide a theoretical foundation for future research and clinical practice while synthesizing recent evidence, identifying unresolved questions, and exploring innovative treatments. Herein, we first examine the immunologic pathogenesis underlying the pathophysiology of AA, as well as the influence of environmental and epigenetic factors, hair follicular cycle disturbances, and genetic susceptibility. Next, we outline current diagnostic and severity assessment tools. We then highlight emerging small‐molecule drugs, biologics, and combination therapies. Finally, we discuss future directions in AA research, emphasizing the need for continued investigation into pathogenesis and targeted therapy development.

## 2 Pathogenesis of AA

**FIGURE 1:**
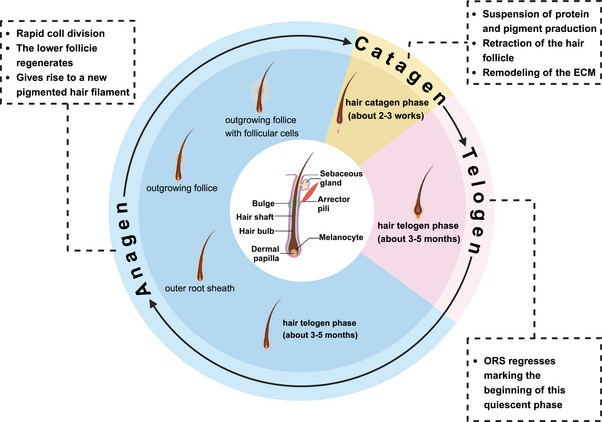
Hair growth cycle. The onset of AA is intricately tied to disruptions in the body's immune system. In typical conditions, the growth of human hair adheres to a cyclical process characterized by distinct phases: growth (anagen), regression (catagen), and resting (telogen). Each hair follicle autonomously follows this cycle. Understanding this complex interplay between immune function and hair follicle dynamics is crucial in comprehending the mechanisms underlying AA and other hair‐related conditions.

The pathogenesis of AA is multifactorial, involving immune dysregulation, genetic susceptibility, environmental factors, and epigenetic alterations. Under normal conditions, hair growth typically follows a cyclical process comprising growth (anagen), regression (catagen), and rest (telogen) phases, with each follicle undergoing an independent cycle (Figure 1). Healthy hair follicles are protected by IP, preventing immune attacks on foreign antigens. However, when this IP is disrupted by specific triggers, self‐antigens are exposed to the immune system, leading to an immune response. This triggers inflammation around the follicle, mediated by IFN‐γ, CD8^+^ T cells, and other immune components, causing the follicle to shift from the anagen to the telogen phase and resulting in hair loss. Genetic predisposition plays a central role in AA, evidenced by familial clustering and associations with specific human leukocyte antigen (HLA) alleles. Genome‐wide association studies (GWAS) have identified multiple susceptibility loci, thereby reinforcing the genetic basis of AA. Environmental factors such as psychological stress, infections, and hormonal fluctuations can exacerbate the condition in genetically vulnerable individuals. Furthermore, epigenetic modifications, including DNA methylation and histone acetylation, dysregulate immune responses and disrupt hair follicle cycling, adding complexity to AA's pathogenesis.

### 2.1 Immune‐Mediated Mechanisms

#### 2.1.1 Immune Cells

CD8^+^ T cells are a crucial subset of cytotoxic T lymphocytes (CTL) within the adaptive immune system, playing a pivotal role in defending against pathogens such as viruses, bacteria, and tumors [30, 31]. Specifically, NKG2D‐expressing CD8^+^ T cells, possessing cytotoxic capabilities, tend to accumulate around the follicular bulb in the affected skin of individuals with AA [32]. CD8 acts as a coreceptor for recognizing peptides presented by MHC‐I class I proteins, while NKG2D is an activating receptor primarily expressed on cytotoxic immune cells. Hence, CD8^+^NKG2D^+^ T cells represent not only specialized CD8^+^ T cells but also a form of genetically engineered chimeric antigen receptor T cells, now recognized as pivotal players in AA's pathogenesis [28]. Studies have demonstrated that CD8^+^NKG2D^+^ T cells alone can induce AA‐like lesions in healthy human skin grafts within SCID mice, leading to elevated levels of these cells in vivo following the onset of the disease [33]. Further investigations in a murine AA model revealed that CD8^+^NKG2D^+^ T cells produce IFN‐γ via the JAK1 and JAK2 pathways, which in turn stimulates the release of IL‐15 by follicular epithelial cells. The production of IFN‐γ is then further stimulated by IL‐15 binding to the surface of CD8^+^NKG2D^+^ T cells via the JAK1 and JAK3 pathways, creating a positive feedback loop [6, 95]. These findings collectively confirm that CD8^+^NKG2D^+^ T cells are sufficient to initiate AA.

CD4^+^ T cells are a specific subpopulation of T lymphocytes that develop in the thymus. These cells express CD4, a glycoprotein on their surface that specifically binds to MHC class II molecules and interacts with lymphocyte‐specific protein tyrosine kinase. CD4 is predominantly found in T cell subsets and thymocytes, with lower expression levels observed in macrophages and DCs [34]. Upon activation by antigen‐presenting cells (APCs), CD4^+^ T cells play a crucial role in regulating other immune cells such as B cells or CD8^+^ T cells and initiating new immune responses [35]. These cells can differentiate into various subtypes, including Th17, Th9, Th2, Th1, regulatory T cells (Tregs), and follicular helper T cells, each producing distinct cytokines to combat pathogens [36, 37]. According to studies, CD4^+^ T cells are present as perifollicular infiltrates in the hair follicles of patients with AA, which may indicate that these cells are involved in the pathophysiology of disease [32, 38]. Experiments in which CD4^+^ T cells from AA‐affected mice were injected subcutaneously into C_3_H/HeJ mice induced generalized alopecia in the recipients. Similarly, research on the Dundee experimental bald rat model of AA has identified a potential effector role of CD4^+^ T cells in alopecia induction [39, 40]. Notably, while both CD4^+^ and CD8^+^ T cells can trigger AA in mice, they contribute differently to the disease: CD8^+^ T cells induce localized AA, while CD4^+^ T cells lead to systemic AA [39]. In summary, the presence and function of CD4^+^ T cells are closely associated with AA, highlighting their significance in the disease process.

Invariant NK T cells (iNKT cells), a subtype of type 1 NKT cells, are unconventional T lymphocytes that recognize lipid antigens such as α‐galactosylceramide (α‐GalCer) presented by CD1d molecules [41]. iNKT cells are 8‐ to 10‐fold larger than both NK cells and conventional T cells. They are abundant in killer cytokines, capable of recognizing specific antigens, and play a crucial role in immune regulation by bridging innate and adaptive immune responses [42]. With both T‐cell receptors (TCRs) and NK‐cell receptors on their surface, iNKT cells exhibit characteristics of both T cells and NK cells. Research has shown that iNKT cells can suppress the progression of AA and promote hair regrowth. In interactions with CD8^+^NKG2D^+^ T cells, iNKT cells have been found to halt the advancement of AA lesions by α‐GalCer stimulation in an animal model with human scalp skin xenografts [42]. These findings position iNKT cells as key modulators of AA pathogenesis and highlight their potential as therapeutic targets for hair regeneration.

Tregs constitute a vital subset of T cells with potent immunosuppressive capabilities, distinguished by the expression of forkhead box P3 (Foxp3), CD25, and CD4 as defining cellular markers. They serve a crucial role in maintaining immune homeostasis within the body [43]. Dysregulation of Tregs‐whether through numerical deficiency or functional impairment‐is implicated in the breakdown of immune tolerance, a hallmark of autoimmune disorders including AA [44]. Although some studies have linked Tregs to AA pathogenesis, showing that IL‐2, IL‐10, and transforming growth factor‐β (TGF‐β) secreted by Tregs can suppress CD8^+^NKG2D^+^ T cells and reduce autoantigen production by hair follicle epithelial cells, these findings remain inconclusive and warrant further investigation [45].

Tissue‐resident memory (TRM) T cells constitute a distinct subset of T cells that permanently inhabit tissues, poised to combat pathogens that may reinfect peripheral tissues [46]. Markers like CD44, CD49, CD69, and CD103 are enriched on TRM T cells, positioning them as the first line of defense against localized infections [47]. TRM T cells are classified into two subsets: IFN‐γ‐producing TRM1 and IL‐17‐producing TRM17. Upon re‐exposure to antigens, these cells rapidly release IFN‐γ, TNF‐α, and other mediators that may contribute to diseases like AA [48]. Furthermore, recurrent AA typically manifests at the original alopecia site, with T cell clones persisting at the lesion site, hinting at the presence of immune memory underlying the pathogenesis of AA [49]. Studies on AA have revealed an upregulation of CD103^+^CD69^+^ TRM T cells at lesion sites in patients. To summarize, TRM T cells have a substantial impact on the development and recurrence of AA, indicating a strong correlation between TRM T cells and the pathophysiology of this illness.

Dendritic epidermal T cells (DETCs), a specialized subset of γδ T cells, exhibit phenotypic and functional overlap with conventional αβ T cells, including upregulated NKG2D expression and the capacity to secrete cytotoxic and inflammatory cytokines. These shared characteristics suggest that DETCs stimulated by self‐antigens have the potential to participate in the pathogenesis of AA by producing IFN‐γ to promote CD8^+^NKG2D^+^ T cell expression and to trigger the disruption of hair follicle IP [50]. An observational study of hair follicles revealed that DETCs were infrequently present in the skin of healthy individuals but significantly elevated in patients with AA [51]. While these findings provide insight into the role of DETCs in AA, further studies are needed to clarify their exact contribution.

**FIGURE 2:**
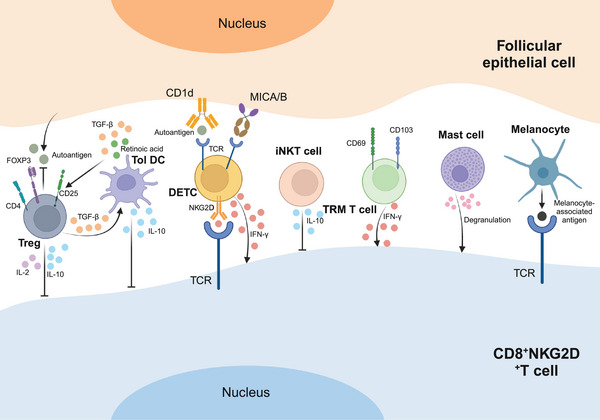
Various cells involved in AA pathogenesis. In the pathogenesis of AA, CD8^+^ NKG2D^+^ T cells play a pivotal role, alongside a spectrum of inflammatory cells including regulatory T cells (Tregs), dendritic cells (DCs), dendritic epidermal T cells (DETCs), natural killer T cells (NKT cells), tissue‐resident memory T cells (TRM T cells), mast cells, and melanocytes. Their concerted interaction around hair bulbs underscores the complex immunological landscape involved in AA progression and offers insights into potential therapeutic targets.

In addition to T cells, several other cell types contribute to the immunologic pathogenesis of AA. Studies show that individuals with AA experience inflammatory infiltration of mast cells and eosinophils in their skin lesions. Mast cells are primarily located near blood vessels and hair follicles, and their numbers correlate with CD8^+^ T lymphocytes around deep hair follicles. In AA patients, mast cell infiltration in mesenchymal, perivascular, and perifollicular regions is more pronounced than in healthy controls. Through degranulation, cytokine release, and enhanced interaction with CD8^+^ T cells, mast cells exacerbate the inflammatory response in AA [52, 53, 54]. Eosinophils, on the other hand, are typically situated at the edges of enlarged AA lesions and can be present throughout all stages of the disease. They are primarily found around hair follicles during the late regressive and resting phases of AA. Notably, the occurrence of eosinophilic infiltration tends to be higher in patients with diffuse AA [54, 55, 56, 57]. Additionally, melanin‐associated antigens from follicular melanocytes activate CD8^+^NKG2D^+^T cells, leading to their attack. These findings underscore the multifaceted involvement of various cell types in the complex pathogenesis of AA (Figure 2).

#### 2.1.2 Cytokines

IFN‐γ stands as the sole member of the type II IFN family, belonging to the class of secreted glycoproteins with a molecular weight of around 17 kDa. When stimulated by cytokines like IL–12, IL‐15, and IL–18, CD4^+^ T cells, CD8^+^ T cells, γδ T cells, and NK cells produce substantial quantities of IFN‐γ. Additionally, NK T cells, B cells, and APC also generate small amounts of IFN‐γ [58, 59]. IFN‐γ not only activates macrophages and induces the expression of MHC‐II‐like molecules but also serves as one of the most crucial endogenous mediators of immune and inflammatory responses, intimately related with several autoimmune illnesses. Studies have revealed that IFN‐γ signaling primarily occurs through the JAK–STAT pathways for transmission and activation [60]. IFN‐γ is considered a pivotal factor in the breakdown of the hair follicle IP system. This process involves the recruitment of CD8^+^NKG2D^+^ T cells to surround the hair follicle, triggering an increase in the expression of cellular MHC‐I‐like molecules. Subsequently, an inflammatory response is activated, leading to the destruction of the IP of the hair follicle, ultimately resulting in autoimmunity and the development of AA. Research has shown that when exogenous IFN‐γ was injected into female C_3_H/HeJ mice, the treated mice exhibited hair follicle atrophy during the growth phase, contrasting with control mice who rarely displayed such atrophy [61]. In another experiment, skin from C_3_H/HeJ mice with AA was transplanted into mice lacking the IFN‐γ gene and wild‐type mice. The results revealed that 90% of the wild‐type mice developed AA, whereas the IFN‐γ‐deficient mice did not develop the condition [62]. These findings underscore the critical role of IFN‐γ in the pathogenesis of AA, regardless of whether the deficiency is endogenous or the acquisition is exogenous. Furthermore, extensive clinical data have validated these experimental findings. Comparative analysis revealed significantly enhanced IFN‐γ‐responsive gene expression profiles in the affected skin of AA patients compared with normal controls. Moreover, serum IFN‐γ levels were markedly elevated in individuals with AA [28, 63‐65]. In conclusion, IFN‐γ emerges as a key player in the pathogenesis of AA, shedding light on potential therapeutic targets for the management and treatment of this autoimmune disorder.

TNF‐α is a 17.4 kDa proinflammatory cytokine crucial in systemic immune and inflammatory responses. It is mainly produced by macrophages and monocytes, but also by neutrophils, CD4^+^ T cells, and NK cells. In autoimmune diseases like AA, TNF‐α plays a central role. Multiple studies have consistently demonstrated elevated serum TNF‐α levels and increased TNF mRNA expression in peripheral blood mononuclear cells among AA patients compared with healthy individuals [66, 67]. Notably, patients with atopic AA exhibited even higher serum TNF levels than those without atopic AA [68]. Further investigations revealed a significant association between serum TNF‐α levels and disease severity, as evidenced by higher TNF‐α levels in AA patients with a Severity of Alopecia Tool (SALT) score of ≥25% compared with those with a SALT score of <25% [69, 70]. Furthermore, there is a positive association between the length of the disease and TNF‐α expression in peripheral blood mononuclear cells [71]. Collectively, these findings underscore the intricate involvement of TNF‐α in the development and progression of AA.

IL‐17 is a key group of proinflammatory cytokines, consisting of six members with similar structures: IL‐17A, IL‐17B, IL‐17C, IL‐17D, IL‐17E, and IL‐17F. These cytokines are primarily synthesized by various immune cells such as CD4^+^ T cells, CD8^+^ T cells, neutrophils, NK cells, and γδ T cells [72]. IL‐17 promotes inflammation by recruiting immune cells, and its effects are amplified when coexists with other proinflammatory cytokines [73]. Notably, IL‐17 has emerged as a pivotal player in AA. Studies have revealed the presence of IL‐17‐secreting cells across all AA types, with a higher prevalence observed in multiple patchy alopecia and a lower occurrence in AT, predominantly localized in the periphery of the hair follicle [74]. Furthermore, elevated levels of Th17 cells and IL‐17 have been consistently observed in AA patients, with a direct correlation noted between serum IL‐17A, IL‐17E, and IL‐17F levels and the severity of the condition [66, 70, 75, 76]. While the roles of other IL‐17 family members in AA remain unclear, it is evident that IL‐17A, IL‐17E, and IL‐17F significantly influence AA pathogenesis.

IL‐2, a key immune system regulator with a molecular weight of 15.5 kDa and a single‐chain polypeptide structure, is primarily secreted by CD4^+^ T cells after activation via TCRs and CD28 costimulatory signaling. It can also be produced by CD8^+^ T cells, NK cells, NKT cells, and DCs [77]. The development of AA has been found to be intricately linked with IL‐2. Heterozygous mice deficient in IL‐2 exhibit reduced expression of IL‐2, IL‐4, IL‐10, IL‐12, IFN‐γ, TNF‐α, and TGF‐β [78]. Additionally, skin grafts from AA‐affected C_3_H/HeJ mice to IL‐2‐deficient mice demonstrated a relative slowing of AA progression [28]. Numerous studies have shown that individuals with AA have higher serum IL‐2 levels than healthy controls, and that their peripheral blood mononuclear cells have higher levels of IL‐2 mRNA [63, 66, 75]. As well, it was discovered that the number of bald plaques on the scalp, the degree of alopecia, and the length of the condition were all positively connected with serum IL‐2 levels in AA patients [63, 75]. However, some studies have suggested that only patients with generalized AA exhibit elevated serum IL‐2 levels compared with healthy controls, while patients with localized AA show no significant changes in serum IL‐2 levels [79]. In conclusion, IL‐2, as a crucial immune cytokine, plays an equally significant role in the pathogenesis of AA.

IL‐15 is a strong proinflammatory cytokine, which structurally identical to interleukin‐2. It is involved in inflammation and autoimmune illnesses in a variety of complex ways [80, 81, 82]. In addition to monocytes and macrophages, other cell types such as fibroblasts, keratinocytes, mast cells, neuronal cells, and DCs can produce this cytokine. A comprehensive transcriptional analysis of AA lesions in both human subjects and C_3_H/HeJ mice revealed upregulation of the IL‐15 gene in affected individuals, and blockade of the IL‐15β receptor significantly decelerated the progression of AA [28]. These findings strongly implicate the upregulation of IL‐15 in the pathogenesis of AA. Further studies have shown increased expression of IL‐15 and its receptor subunit IL‐15Rα in the hair follicles of AA patients, who also exhibit elevated serum IL‐15 levels [83, 84]. Additionally, a positive correlation exists between serum IL‐15 levels and the severity of AA. Collectively, these findings establish IL‐15 as a pivotal cytokine in the pathogenesis of AA, serving as a critical signaling molecule in the development of this condition.

IL‐12 is a heterodimer composed of an α‐subunit (IL‐12p35) and a β‐subunit (IL‐12p40), with the latter being shared structurally with IL‐23. IL‐12 exhibits its activity only when both subunits are present [85]. This proinflammatory cytokine is primarily produced by DCs, macrophages, and B cells in response to stimulation by microbial pathogens [86]. Studies have shown that patients with AA have significantly higher serum IL‐12 levels and increased expression of IL‐12 mRNA in peripheral blood mononuclear cells compared with healthy controls [67, 68, 87]. Furthermore, IL‐12 levels in peripheral blood mononuclear cells have been found to positively correlate with the severity and duration of hair loss [71]. As a result, it is believed that IL‐12 plays a significant role in the pathophysiology of AA.

Patients with AA show a significant presence of Th1 cytokines (such as IL‐2, IFN‐γ, TNF‐α, IL‐12, and IL‐18) and Th2 cytokines (including IL‐4, IL‐5, IL‐6, IL‐9, IL‐10, IL‐13, IL‐17E, IL‐31, and IL‐33), and Th17 cytokines (such as IL‐17, IL‐17F, IL‐21, IL‐22, and IL‐23), which contribute to the condition. However, various other cytokines also play a critical role in maintaining hair follicle IP and restoring collapsed IP [88] (Table 1). These additional cytokines can be likened to “follicular IP security guards” as they protect hair follicles from immune damage. For example, IL‐10 regulates T‐cell secretion and prevents T‐cell proliferation and inflammatory cytokine production, which helps preserve the immunosuppressive environment in the hair follicular IP [89]. Similarly, TGF‐β, a key cytokine for immune regulation and homeostasis, downregulates MHC‐I expression in T cells, inhibiting the onset of AA [17, 42, 90]. Furthermore, factors such as calcitonin gene‐related peptide (CGRP), α‐melanocyte‐stimulating hormone (α‐MSH), Substance P (SP), vasoactive intestinal polypeptide (VIP), macrophage migration inhibitory factor (MIF), indoleamine2,3‐dioxygenase (IDO), thrombospondin 1 (TSP1), and IK cytokine (Red/IK) are also involved in inhibiting the expression of MHC‐I classes through immune regulation, thereby serving a similar purpose [17, 91‐93].

**TABLE 1:** Cytokines associated with AA.

Typology	Cytokines	Variations	Acceptor	Primary target cells	Function
Th1‐type	IFN‐γ [64, 65, 68]	Raise	IFNGR1/IFNGR2	Th1 cells, CTL cells, NK cells, DCs, macrophages, epithelial cells (almost all cells except erythrocytes)	Destruction of hair follicles IP
	IL‐2 [68, 94, 95]		CD25/IL2RA, CD122/IL2RB, CD132/IL2RG	CD4^+^ and CD8^+^ activated T cells, NK cells, B cells	
	IL‐12 [67, 89]		CD212/IL12RB1, IR12RB2	Activated T cells (mainly Th1 cell), NK cells	
	IL‐18 [95]		CDw218a/IL‐18R1	Th1 cells, NK cells, macrophages, etc.	
Th2‐type	IL‐4 [79, 89]	Raise	CD124/IL4R, CD132/IL2RG	Activated B cells, T cells	
	IL‐5 [96]		CD125/IL5RA, CD131/IL3RB	Eosinophils, basophils, mast cells	
	IL‐6 [95, 97]		CD126/IL6RA, CD130/IL6RB	T cells, B cells	
	IL‐13 [63, 98, 99]		IL13R	Th2 cells, B cells, macrophages	
	IL‐25 [76]		IL17R	Th2 memory cells	
	IL‐33 [76]		ST2	Eosinophils, basophils, NK cells, NKT cells, Th2 cells, DCs	
	IL‐9 [68, 99]		CD129/IL9R	Th9 cells, mast cells, keratinocytes	
Th17‐type	TNF‐α [67, 68]	Raise	CD120a,	CD4^+^ T cells, mast cells, neutrophils, NK cells	
	IL‐17 [75, 76]		CDw217/IL17RA, IL17RB	Epithelial tissue cells, endothelial tissue cells	
	IL‐21 [70, 76]		IL21R	CD4^+^ T cells, CD8^+^ T cells, B cells, DCs, macrophages, keratinocytes	
	IL‐23 [76, 100]		IL23R	T cells (mainly Th17 cells), macrophages	
	IL‐22 [45, 87]	No significant changes	IL22R	Keratin‐forming cells, subepithelial myofibroblasts	
Immunomodulatory factor	SP [101]	Raise	NK1R	T cells, monocytes, macrophages, eosinophils, neutrophils, mast cells	
	IL‐15 [102]	Raise	CD122/IL‐15R	CD8^+^ T cells, DCs, NK cells, mast cells,	
	IL‐10 [63, 76, 96]	No significant changes	CD210/IL10RA, CDw210B/IL10RB	Macrophages, B cells, mast cells, Th1 cells, Th2 cells	Maintains follicle IP
Regulatory T‐cell class	TGF‐β [63, 66, 90]	Decrease	TGFβR	Wide expression	
Neurohormone class	CGRP [103, 104]	Decrease	RAMP1	Neutrophils, monocytes, macrophages	
	VIP [105]		VIPR	Astrocytes, microglia, and peripheral inflammatory cells	
	α‐MSH [106]	Raise	MC1R, MC5R	Melanocytes, bone marrow cells	
other	MIF [107]	Not yet sure	CXCR2, CXCR4, CXCR7, CD44, CD74 complexes	Monocytes, macrophages	
	IDO		CD25	DCs, monocytes, macrophages	
	TSP1		CD36, CD47	Vascular smooth muscle cells, endothelial cells, fibroblasts, inflammatory cells, and macrophages	
	PD‐L1	Decrease	PD‐1	Activated T cells, B cells, DCs, keratinocytes, and monocytes	
	Red/IK		Indefinite	Indefinite	

Abbreviation: PD‐L1: programmed death‐ligand 1.

#### 2.1.3 Signaling Pathways

Multiple inflammatory signaling pathways are involved in the pathogenesis of AA. As previously mentioned, the Th1 pathway‐related cytokines IFN‐γ and TNF‐α, as well as the Th17 pathway signature cytokine IL‐17A, were found to be significantly elevated in the sera of AA patients. The degree of inflammation in AA was also significantly and positively correlated with the levels of these cytokines. This not only highlights the importance of cytokines secreted by immune cells in the development of AA, but also indicates the involvement of Th1 and Th17 inflammatory signaling pathways in its pathogenesis [75, 108, 109]. Despite the general consensus that AA is a type 1 inflammatory autoimmune disease mediated by the Th1 and Th17 pathways, patients with AA have been shown to have higher serum and scalp levels of Th2‐associated biomarkers such IL‐4. These results imply that Th2‐associated cytokines might be involved in the AA pathogenesis as well [83, 110]. In conclusion, the pathogenesis of AA is complex and involves multiple inflammatory signaling pathways, including Th1, Th2, and Th17 [111].

In recent years, the JAK–STAT signaling pathway has emerged as a focal point in AA research. This intracellular pathway is pivotal in regulating cytokine levels and immune responses, and comprises cytokine receptors, JAK, and STAT as its three key components. The JAK family encompasses cytoplasmic tyrosine kinases, including JAK1, JAK2, JAK3, and TYK2. JAK1 primarily mediates signals associated with inflammatory diseases, while JAK2 is principally involved in signaling related to red blood cell and platelet production. Signals mediated by JAK3 are predominantly associated with autoimmune diseases [112]. The STAT family of proteins is distinctive in their ability to bind to DNA and function as substrates and downstream signaling molecules for JAK kinases. It consists of the transcription factors STAT1, STAT2, STAT3, STAT4, STAT5a, STAT5b, and STAT6, which mediate distinct intracellular signaling pathways related to immunological homeostasis, cell proliferation, differentiation, and organ development. Modulating different JAK–STAT pathways can have diverse effects on signaling. Indeed, existing studies have demonstrated that the JAK–STAT pathway plays a central role in regulating most autoimmune diseases.

**FIGURE 3:**
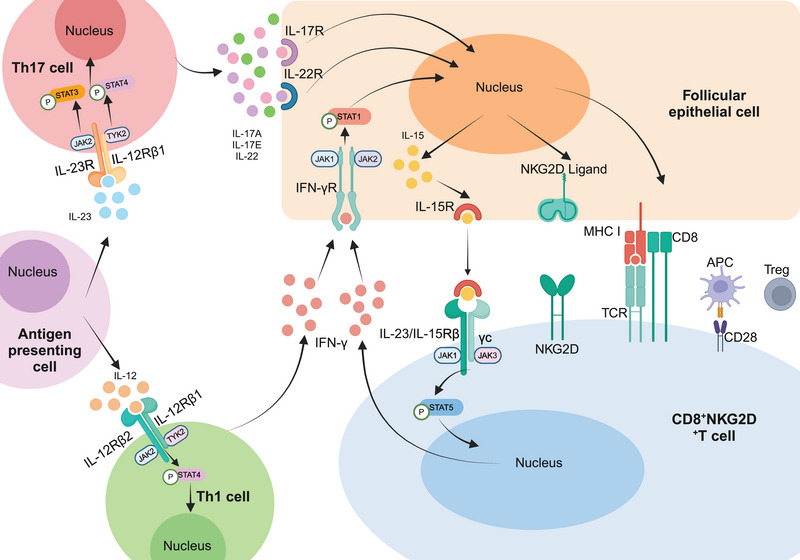
JAK–STAT pathway. In AA, stimulatory factors activate CD8^+^ NKG2D^+^ T cells, inducing IFN‐γ production through the JAK1 and JAK3 pathways. IFN‐γ, in turn, enhances IL‐15 production in follicular epithelial cells via JAK1 and JAK2 signaling. IL‐15 binds to CD8^+^ NKG2D^+^ T cells, further stimulating IFN‐γ production, thereby amplifying this positive feedback loop. In addition, binding of IL‐23 and IL12 to their respective receptors activates the JAK2–STAT3 pathway, the TYK2–STAT4 pathway, and the JAK2–STAT4 pathway, respectively. This leads to the secretion of large amounts of IL‐17A, IL‐17F, and IL‐22 by Th17 cells and the release of large amounts of IFN‐γ by Th1 cells.

AA, as a representative autoimmune skin disease, is intricately linked to the JAK–STAT pathway in its pathogenesis. While the specific mechanism behind AA remains complex and elusive, a significant proportion of cytokines implicated in its pathogenesis lack intrinsic kinase activity in their receptors. Consequently, these cytokines rely on signaling through the JAK–STAT pathway, making it a prominent focus in current studies of AA pathogenesis [113]. In the context of AA, the activation of CD8^+^NKG2D^+^ T lymphocytes by cytokines via JAK1 and JAK3 leads to the substantial production of IFN‐γ. This cytokine binds to receptors on the surface of follicular epithelial cells, triggering the JAK1/2–STAT1 pathway, thereby augmenting the production of IL‐15 by follicular epithelial cells. Subsequently, the binding of IL‐15 to the receptor on the surface of CD8^+^NKG2D^+^ T cells activates the JAK1/3–STAT5 pathway, perpetuating the production of IFN‐γ. This creates a positive feedback loop that amplifies the inflammatory response, exacerbating the condition (Figure 3). Furthermore, the IL‐23p19 subunit of IL‐23 and the IL‐12/IL‐23p40 subunit of IL‐23 trigger the activation of the JAK2–STAT3 pathway and the TYK2–STAT4 pathway, respectively, upon binding to their respective receptors. This activation leads to the secretion of significant amounts of IL‐17A, IL‐17F, and IL‐22 by Th17 cells. Additionally, the p35 subunit of IL‐12 and the shared IL‐12/23p40 subunit of IL‐12 activate the TYK2–STAT4 pathway upon receptor binding, as well as the JAK2–STAT4 pathway, resulting in the release of abundant IFN‐γ by Th1 cells. Apart from these cytokines, molecules like IL‐2, IL‐7, and IL‐21 also target the epithelial cells of hair follicles, accelerating the follicles' progression into the degenerative phase, ultimately leading to hair loss [33, 114, 115] (Figure 3).

### 2.2 Environmental and Epigenetic Factors

#### 2.2.1 Environmental Factors

The intricate pathogenesis of AA involves a close interplay between epigenetic mechanisms and environmental factors. Among environmental triggers, psychological stress and lifestyle habits significantly influence disease development. Chronic exposure to mental stress, anxiety, or depression can lead to abnormal activation of the hypothalamic–pituitary–adrenal axis, resulting in sustained cortisol elevation [116]. This hormonal dysregulation not only suppresses hair follicle stem cell activity but also disrupts immune equilibrium through dual pathways: compromising the IP of hair follicles while stimulating proinflammatory cytokine release, thereby exacerbating immune dysfunction and ultimately causing hair loss [117]. Epidemiological evidence reinforces this mechanism, with clinical data showing that nearly 80% of patients experienced major psychological trauma or persistent anxiety within 6 months prior to disease onset, underscoring the critical role of environmental stress in AA initiation.

Multiple factors in the living environment are likewise involved in the disease process [118, 119]. The data show tobacco exposure exhibits a positive correlation with AA risk, potentially mediated by smoke‐induced oxidative stress responses and dominant expression of proinflammatory cytokines [120]. Notably, alcohol consumption demonstrates bidirectional effects‐moderate intake may alleviate stress, whereas excessive use worsens immune dysregulation [118]. Certain medical interventions, including hepatitis B vaccination, highly active antiretroviral therapy, and amphetamine administration, have been identified as potential triggers [121, 122, 123]. Dietary patterns also play a role: deficiencies in micronutrients (vitamins, zinc, selenium, folate) and proteins directly impair hair follicular physiology, while modern dietary excesses aggravate pathology through multiple mechanisms [124, 125, 126, 127, 128, 129]. High‐fat diets reduce microcirculatory efficiency and disrupt sex hormone balance, whereas excessive sugar intake activates insulin signaling to induce chronic inflammation. These metabolic disturbances collectively interfere with normal hair follicular function, potentially triggering AA onset. Consequently, targeted lifestyle modifications‐encompassing stress reduction, smoking cessation, balanced nutrition, and alcohol moderation‐emerge as vital strategies for both prevention and clinical management of this condition.

#### 2.2.2 Epigenetic Factors

In recent years, the role of epigenetic regulatory mechanisms in the pathogenesis of AA has gained increasing attention [130]. Epigenetics refers to the regulation of gene expression through chemical modifications without altering the DNA sequence. In AA, these modifications including DNA methylation, histone modifications, and microRNAs (miRNAs) can regulate the expression of key immune response genes, thereby contributing to disease initiation and progression [131, 132]. Current research focuses on identifying epigenetic alterations in AA patients, which are closely linked to disease mechanisms. Studies have revealed abnormal DNA methylation patterns in AA patients, along with dysregulated expression of epigenetic regulators such as methyl‐CpG binding domain protein 1, DNA‐methyltransferase 1 (DNMT1), and histone deacetylase 2 (HDAC2) [133]. Distinct miRNA profiles have also been identified: miR‐1246 and miR‐210 show potential as diagnostic biomarkers to differentiate AA patients from healthy controls, while miR‐185‐5p, miR‐125b‐5p, and miR‐186‐5p are upregulated in patients with severe, active disease [130]. In C3H/HeJ mouse models of AA, lesions exhibit significant overexpression of mmu‐miR‐155 alongside downregulation of mmu‐miR‐1, mmu‐miR‐101a, and mmu‐miR‐705 [134].

Epigenetics serves as a critical bridge connecting environmental factors to gene expression, modulating immune system activity and driving autoimmune attacks against hair follicles. Emerging evidence suggests viral infections—such as cytomegalovirus, human papillomavirus, Epstein–Barr virus, human immunodeficiency virus (HIV), hepatitis B/C viruses, and severe acute respiratory syndrome coronavirus 2—may indirectly participate in AA pathogenesis or progression through epigenetic reprogramming [121, 135‐137]. These pathogens have been detected prior to AA onset, potentially altering host epigenomes to disrupt immune tolerance.

In summary, epigenetic research offers novel therapeutic perspectives for AA. By targeting specific epigenetic marks, it may be possible to restore normal gene function, halting or reversing disease progression. The epigenetics of AA represents a complex and evolving field, deepening our understanding of its pathogenesis and revealing potential therapeutic targets for future interventions.

### 2.3 Hair Follicle Cycle Disruption

As previously discussed, AA is an autoimmune disease primarily driven by aberrant immune attacks targeting hair follicles. However, dysregulation of the hair follicle cycle also constitutes a critical pathological component in AA pathogenesis. This cycle disruption manifests as impaired transition between growth phases, characterized by shortened anagen phase, premature catagen phase onset, and compromised regenerative capacity, ultimately leading to hair loss.

It is well known that hair follicles are skin appendages composed of mesenchymal and epithelial components, with a complex structure, unique stem cell niche, and self‐renewal ability [138]. Additionally, hair follicles are immune‐privileged organs that ensure continuous hair growth and periodic regeneration of the follicle [139]. The normal hair follicle cycle is dynamically regulated by the anagen, catagen, and telogen phases. Under normal conditions, the duration of each phase and the proportion of follicles in each phase are relatively stable. During the anagen phase, hair follicles maintain the proliferation of matrix cells through insulin‐like growth factor 1 (IGF‐1), vascular endothelial growth factor (VEGF), and activation of the Wnt/β‐catenin signaling pathway secreted by dermal papilla cells (DPCs). During the catagen and telogen phases, follicle regression and quiescence are regulated by apoptotic molecules (such as Bax, Fas) and cell cycle inhibitors (such as p21, p27). However, in AA patients, IFN‐γ binds to the receptor on hair follicle keratinocytes, activating the JAK–STAT signaling pathway, which releases a large number of proinflammatory factors (such as TNF‐α) and induces the expression of chemokines (such as CXCL9/10/11), further recruiting immune cells and forming a localized inflammatory microenvironment. This process not only exacerbates immune attack but also directly damages anagen hair follicle epithelial cells and inhibits the differentiation ability of hair follicle stem cells, triggering hair follicle cycle disruption. This is manifested by a significant shortening of the anagen phase (from the normal 2–6 years to a few weeks to months), premature initiation of the catagen phase, and prolonged telogen phase, creating a “rapid degeneration‐delayed regeneration” vicious cycle.

It is noteworthy that oxidative stress plays a role as an amplifier in this process. Oxidative stress may influence the hair follicle microenvironment, leading to hair follicle cycle disruption and thus contributing to the onset and development of AA. Studies have shown that excessive accumulation of reactive oxygen species induces abnormal expression of NKG2D ligands (MICA/ULBP), which activates NK cells and exacerbates immune attack; at the same time, it significantly decreases the activity of antioxidant enzymes such as superoxide dismutase and glutathione peroxidase, elevates lipid peroxidation products like malondialdehyde, and creates a pro‐oxidative microenvironment [16, 140, 141]. Recent single‐cell sequencing studies have revealed that with hair follicle cycle disruption, the number of immune subsets such as macrophages, mast cells, and NK cells dynamically changes, highlighting the interaction between immune attack and hair follicle cycle disruption in the pathogenesis of AA [142, 143, 144, 145]. Additionally, abnormalities in epigenetic regulation, such as DNA methylation dysregulation (e.g., DNMT1 imbalance) or miRNA network imbalance (e.g., overexpression of miR‐155), can transform environmental stressors (such as psychological stress or viral infections) into persistent drivers of hair follicle cycle disruption.

In conclusion, hair follicle cycle disruption is the core mechanism of AA's pathophysiology, involving immune attacks that directly damage the structure of anagen hair follicles, instability of the stem cell niche, oxidative stress, and collaborative damage from epigenetic modifications. This theoretical framework provides guidance for the development of new therapeutic strategies: targeting key nodes in the cycle regulation (such as Wnt pathway activators), intervening in the immune‐hair follicle interface (such as JAK inhibitors), and reconstructing redox balance. These combined treatment approaches may break through the current treatment bottleneck and promote the development of precision medicine.

### 2.4 Genetic Predisposition

AA is associated with various factors, including immune system abnormalities, environmental influences, and genetic factors. Studies have shown that AA has a significant genetic predisposition, with many cases having a family history of the disease [146]. However, the genetic susceptibility to AA is not a simple Mendelian inheritance pattern but rather a complex, polygenic regulatory process [135, 147, 148].

Genetic studies have found that AA is linked to variations in multiple gene loci, particularly those related to the immune system. Among them, the HLA gene cluster is closely associated with susceptibility to AA, especially certain subtypes of HLA‐DQ and HLA‐DR, which increase the risk of developing the condition [149]. Furthermore, GWAS have revealed several genes associated with AA, including those related to immune regulation and hair follicle cycle. In total, 14 genomic regions have been identified as related to the disease, such as genes involved in IFN‐γ production, NKG2D‐mediated cytotoxicity (ULBP3/6, MICA, and IL2), T cell activation and proliferation (CTLA‐4, IL2, IL21, IL2RA, SOCS1, IKZF4/Eos, GARP/LRRC32, SH2B3(LNK)/ATXN2, IL23A, PTPN22, CD28, ICOS, and IL13), HLAs (HLA‐DRB1, C6orf10, BTNL2, HLA‐DRA, HLA‐DQA1, HLA‐DQA2, and HLA‐DQB2), as well as hair follicle‐related genes (STX17, PRDX5, ACOXL/BCL2L11, ERBB3, and CCHCR1) [149, 150, 151, 152, 153]. Many of these genes, such as CTLA‐4, ICOS, and TCR genes, are thought to be associated with genetic susceptibility to AA.

In addition, a genome‐wide copy number variation analysis of candidate genes revealed duplications of melanocortin receptor 2 (MCHR2) and its antisense RNA (MCHR2‐AS1), suggesting the involvement of genes affecting pigmentation [154]. This finding may explain why hair regrowth in AA patients often turns white after an acute flare‐up. Variations in other related genes, such as syntaxin‐17 (STX17) and peroxiredoxin‐5 (PRDX5), indicate a potential role of oxidative stress in the pathogenesis of AA [155]. The discovery of coil‐coiled α‐helix protein 1 (CCHCR1) suggests that keratinization disorders may also be linked to the pathogenesis of AA [153]. The interaction between genetic and environmental factors is also an important component of genetic susceptibility to AA. Also, certain environmental factors, such as infections and stress, can trigger AA in people with a genetic predisposition. In conclusion, genetic susceptibility plays a significant role in the pathogenesis of AA, and family history is an important risk factor.

## 3 Diagnosis and Evaluation of AA

AA has a variety of clinical subtypes, which can primarily be identified and categorized essentially according to these features [156, 157].

**FIGURE 4:**
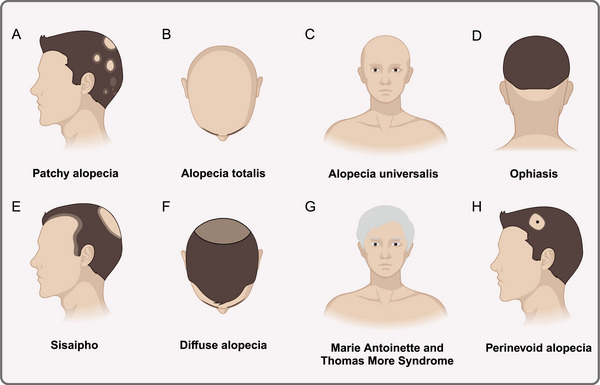
Clinical manifestations of AA. (A) Patchy alopecia; (B) Alopecia totalis; (C) Alopecia universalis; (D) Ophiasis; (E) Sisaipho; (F) Diffuse alopecia; (G) Marie Antoinette and Thomas More syndrome; (H) Perinevoid alopecia.

The most prevalent kind of alopecia is patchy alopecia, which manifests as one or more distinct, nonscarring bald patches that are round or oval in shape and vary in size and position. The damaged scalp has a smooth, sometimes pink surface. The majority of patients have no symptoms, however others, particularly prior to the disease's beginning, experience localized tingling, itching, or unusual sensations (Figure 4A); complete or nearly total loss of scalp hair is a symptom of AT (Figure 4B). The condition known as AU is characterized by the nearly complete loss of all body hair, including pubic, axillary, beard, eyebrow, and cuirass hair (Figure 4C); ophiasis is characterized by a symmetrical band of hair loss along the occipital hairline that extends sinuously to the bi‐temporal area (Figure 4D). Treatment for this condition is challenging and the prognosis is uncertain; sisaipho is characterized by a large loss of hair in the middle of the scalp and a less noticeable loss of hair around the edges (Figure 4E); diffuse alopecia extends across the entire scalp, but tends to be diffuse and does not involve all of the hair (Figure 4F). The symptoms of Marie Antoinette and Thomas More Syndrome include an abrupt “overnight white head” phenomena and significant color‐bearing hair loss in a brief period of time [158]. This is because white‐haired hair follicles may survive on the scalp while pigment‐producing hair follicles are more vulnerable to inflammatory cells' selective attack (Figure 4G). One of the less common forms of AA is perinevoid alopecia, in which patches of baldness typically encircle pigmented naevi, much like halo nevi (Figure 4H). Another very uncommon form of AA is called AAI, which is more common in women and is characterized by diffuse hair thinning rather than patchy alopecia, no nail involvement, and a typically quick onset that lasts for months to years [159, 160]. AAI is reported to be characterized by a number of yellow and black dots, regrowth of short cui‐ui hairs, dystrophic hairs, and hairs that resemble exclamation points [161]. With only about 100 documented cases of AAI, there is an absence of the practical basis for ensuring an accurate diagnosis of the disease. Some researchers have categorized it as Diffuse alopecia.

Currently, the diagnosis and evaluation of AA is mainly based on clinical manifestations and a series of auxiliary examinations, including hair‐pulling test, dermoscopy, laboratory examination, histopathologic examination of skin lesions, etc., and basically do not need to carry out special examinations.

### 3.1 Clinical Manifestations of AA

In clinical presentation, patients with AA generally do not exhibit any abnormal skin appearance, but they develop one or more well‐defined, round or oval patches of hair loss on the scalp. These patches typically measure between 1 and 2 centimeters in diameter, although they can vary in size. Hair loss may occur anywhere on the scalp and can also affect other areas, such as the eyebrows and eyelashes [4]. The affected areas are usually free of inflammation, scaling, and scarring. The edges often show loose, easily shed hairs, some of which may be broken, and the hairs at the proximal end tend to be thinner, with a loss of pigment in the lower portion of the hair. As the condition progresses, the affected patches may increase in number and merge, forming irregular shapes, potentially advancing to AT or AU.

Around 10–6% of AA patients may develop nail damage, which is a common complication of the condition [162]. Nail changes in AA patients can manifest in several forms, including superficial nail pitting (affecting about 30% of nails) and rough nails, also known as sandpaper nails (in over 10% of cases) [163]. Other manifestations include white nails, nail detachment, and nail loss [164]. Notably, when acute nail changes such as red spots on the nail arches and periungual redness appear, this suggests that the immune system is attacking healthy nail cells. Additionally, 10–22% of AA patients may have atopic tendencies, 8% to 28% may be affected by autoimmune thyroid disease, and 1–4% may have concurrent vitiligo, there is the risk of diabetes and Down's Syndrome, and so on [165, 166, 167, 168]. Furthermore, AA hair loss typically avoids areas of inflammation, such as lesions associated with psoriasis.

Other hair loss disorders can present with AA‐like features. For example, androgenetic alopecia (AGA) typically involves gradual, diffuse hair loss with broader areas of involvement; trichotillomania (hair‐pulling disorder) usually presents as irregular, patchy hair loss with uneven edges, and hair in the affected areas may not be completely lost; tinea capitis (scalp ringworm) often presents with red patches, scaling, and crusting, and fungal elements can be seen in the lesions; scarring alopecia results in permanent localized hair loss due to various causes, with visible scalp atrophy, scarring, or sclerosis; syphilitic alopecia shares similar dermoscopic and histopathological features with AA, but serum syphilis‐specific antibodies are positive, and clinical signs may include “moth‐eaten” patches of hair loss. In such cases, it is essential to use the Alopecia Areata Assessment Tool to differentiate AA from other conditions, including trichotillomania, tinea capitis, scarring alopecia (lichen planopilaris, frontal fibrosing alopecia), syphilitic alopecia, telogen effluvium, female‐pattern AGA, and congenital alopecia, in order to accurately diagnose and select appropriate treatment for AA patients.

### 3.2 Supportive Diagnosis of AA

#### 3.2.1 Hair Pulling Test

The hair pull test is a simple but effective method of diagnosing AA. The patient does not shampoo for 3–5 days, then pulls up a bundle of about 50 or 60 hairs with the thumb and forefinger and gently pulls it down the hair shaft toward the end of the hair, if more than six hairs are pulled off, it is positive and suggests the possibility of spot AA.

#### 3.2.2 Dermoscopy

Dermoscopy is a noninvasive test that is important in the diagnosis, identification and evaluation of AA [169]. Typical AA is characterized by the “exclamation mark” of the hairs (thinning in the mid‐section of the hairs and thickening at the ends), as well as black dots and changes in the opening of the hair follicles in the area of hair loss. If the dermoscopic performance of the hair loss area is observed and yellow dot sign, black dot sign, broken hair, dystrophic hair, conical hair, exclamation mark‐like hair, short vellus hair, and so on are found, then the diagnosis of AA can be confirmed. Apart from this, dermoscopy also helps in identifying other types of hair loss.

#### 3.2.3 Histopathological Examination

In cases where the diagnosis is difficult to make, histopathological examination may be performed. The histopathological picture of AA shows a peripheral inflammatory cellular infiltrate around the bulb of the hair, mainly lymphocytes with a few eosinophils and mast cells. (The immune cells associated with AA have already been mentioned above.) In addition, follicular miniaturization and dystrophic anagen follicles were seen, especially in patients with progressive and recovering AA. The diagnosis of AA can be further confirmed by observing these morphological and structural changes in the hair follicles [57].

#### 3.2.4 Laboratory Tests

The diagnosis of most AAs relies primarily on clinical manifestations, but laboratory tests may be performed to rule out other immune, allergic, and systemic diseases [169]. For example, thyroid function, anemia, and immunological markers (e.g., antinuclear antibodies, antibody titers, etc.) can be checked to help identify complications of AA represented by thyroid disease, anemia, or other autoimmune diseases.

#### 3.2.5 Genetic Testing

In recent years, as genomics continues to evolve and develop, some researchers have begun to explore the possibility of genetic testing to aid in the diagnosis of AA. They have found that some genes (e.g., HLA genes, CTLA‐4 genes, ULBP1 genes, etc.) associated with immune system function, hair follicle biology, and inflammatory pathways are somehow related to AA [170, 171]. By assessing changes in these genes, it may be possible to more accurately diagnose AA or predict a patient's response to treatment. Although genetic testing has not yet become a routine clinical diagnostic tool, it can be used as a supplement to clinical diagnosis and provide some theoretical basis for studying the etiology and genetic susceptibility of AA.

### 3.3 Evaluation of AA

#### 3.3.1 SALT Scores

SALT, as specified in the American Alopecia Areata Assessment Guidelines, has been widely used in clinical practice to assess the severity and progression of AA [172, 173]. This scoring system divides the scalp into four regions, each corresponding to a percentage of the total scalp area: the left side (18%), right side (18%), top (40%), and back (24%) [173]. By visually estimating the degree of hair loss in each region and summing these percentages, the final SALT score is determined. According to the system, the scores are classified into six categories: S0 (no hair loss), S1 (less than 25% hair loss), S2 (25–49% hair loss), S3 (50–74% hair loss), S4 (75–99% hair loss), and S5 (100% hair loss). The severity of AA is typically classified as follows: mild AA when <25% of the scalp area is affected, moderate AA when 25–49% is affected, and severe AA when ≥50% is affected.

To provide a more comprehensive evaluation, the SALT scoring system was expanded to include assessments of hair loss outside the scalp (B) and nail involvement (N). The “B” category evaluates body hair loss: B0 indicates no hair loss, B1 indicates partial body hair loss, and B2 represents total body hair loss. The “N” category assesses nail involvement: N0 indicates no nail changes, N1 indicates partial nail changes, and N1a refers to all 20 nails showing abnormalities. These additional assessments help provide a fuller picture of the patient's condition, aiding in the formulation of more effective treatment strategies [174].

#### 3.3.2 Scale Assessment Tools

A consensus developed by clinical experts in the United States has established a comprehensive scale for assessing the severity of AA, taking into account factors such as hair loss, involvement of eyelashes and eyebrows, and psychological impact [175]. This scale includes both primary and secondary indicators. The primary indicators assess the extent of hair loss: alopecia is classified as mild when hair loss is less than 20%, moderate when it ranges from 21 to 49%, and severe when it reaches 50–100%. The secondary indicators apply to patients with mild or moderate alopecia and suggest upgrading the severity level if one or more of the following conditions are present: the alopecia significantly affects the patient's psychological or social well‐being, there is substantial involvement of eyebrows or eyelashes, there is no notable improvement after at least 6 months of treatment, or the hair pull test is positive, indicating rapid disease progression.

The psychological impact of AA has become a crucial factor in evaluating its severity. Several widely used tools are available to assess this aspect: the Dermatology Life Quality Index, which evaluates the overall quality of life affected by skin conditions and is known for its ease of use; the Hospital Anxiety and Depression Scale, designed to assess anxiety and depression with 14 items (seven for anxiety and seven for depression); the Perceived Stress Scale (PSS‐14), a self‐report tool for measuring perceived stress levels; and the Plutchik Suicide Risk Scale, which is particularly useful for assessing suicide risk in individuals with suicidal thoughts or a history of depression. These tools play a vital role in understanding the psychological burden faced by AA patients.

#### 3.3.3 Other Evaluation Tools

In clinical practice, the SALT score is a widely used tool for assessing the severity of AA. However, there are several other assessment tools that can complement this evaluation, such as the 16‐item Skin Disease Quality of Life Index, the Appearance of Patches of Alopecia Areata Rating Scale, the Alopecia Areata Severity Index Scale, the Patches of Alopecia Areata Quality of Life Index, and the Work Productivity and Activity Impairment in Alopecia Areata Questionnaire, among others [176]. To gain a more comprehensive understanding of the disease, it is important to consider multiple factors, including the extent of hair loss (not limited to the scalp), patient quality of life, treatment response, disease activity, psychological impact, and treatment needs. By integrating these dimensions, a more complete picture of the patient's condition can be obtained. Ultimately, the goal of assessment is to develop an individualized treatment plan tailored to the patient's specific situation, thereby improving the effectiveness of AA management.

## 4 The Therapy for AA

Current research into signaling pathways involved in the pathogenesis of AA remains limited, with focus primarily on the classical JAK–STAT pathway. While this pathway is well understood, other signaling pathways have yet to be fully explored. This incomplete understanding poses a challenge for developing comprehensive treatments for AA. However, these signaling pathways, along with associated cytokines, hold potential as biomarkers for identifying the pathogenesis of AA and as targets for potential therapeutic interventions.

**FIGURE 5:**
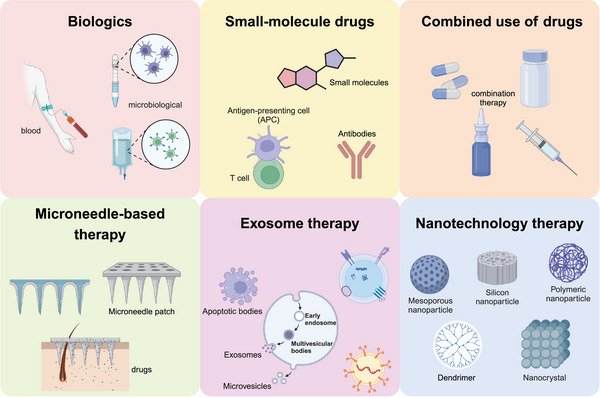
Emerging treatments for AA. Traditional treatments have focused on immune modulation and topical solutions, but recent years have seen a surge in innovative therapeutic approaches. From biologics and small‐molecule drugs targeting specific pathways to synergistic combinations and cutting‐edge techniques such as microneedling, exosome therapy, and nanoparticle delivery systems, the landscape of AA treatment is rapidly evolving. This exploration into emerging therapies not only aims to mitigate hair loss but also holds promise for restoring hair growth in individuals affected by this autoimmune condition.

In recent years, due to the various limitations and shortcomings of traditional therapies, there has been growing interest in emerging treatments for AA (Figure 5). Biologic agents that inhibit pathways such as Th17, Th2, IL‐12/IL‐23, TNF‐α, as well as small‐molecule drugs targeting JAK–STAT and PDE‐4, are progressively advancing toward clinical application (Table 2). This expanding range of treatment options represents a significant enrichment in the management of AA.

**TABLE 2:** Drugs targeted for the treatment of AA.

Treatments	Drugs	Classifications	Targets of action	Indications	Clinical process	Main initiating country	NCT
Biological preparation	Etanercept	TNF inhibitor	TNF‐α	Not yet defined	None	None	Not applicable
	Adalimumab		TNF‐α	Not yet defined	None	None	Not applicable
	Ustekinumab	IL‐12/IL‐23 inhibitor	IL‐12, IL‐23	Severe AA	None	None	Not applicable
	Tildrakizumab	IL‐23 inhibitor	IL‐23	Chronic, severe AA	None	None	Not applicable
	Secukinumab	Th17 signaling pathway inhibitor	IL‐17A	Generalized AA	Terminate	USA	NCT02599129
	Dupilumab	Th2 signaling pathway inhibitor	IL‐4 and IL‐13	Moderate to severe AA; AA in children	Phase II	USA	NCT03359356 NCT05866562
	Tralokinumab		IL‐13	Moderate to severe AA	Phase II	USA	NCT02684097
	Rosnilimab (ANB030)	PD‐1 checkpoint agonist antibody	PD‐1 agonist	Moderate to severe AA	Phase II	USA	NCT05205070
	Daxdilimab (HZN‐7734)	LILRA4(ILT7) inhibitor	LILRA4 (ILT7)	Moderate to severe AA	Phase II	USA	NCT05368103
	Platelet‐rich plasma (PRP)	Plasma concentrates	TGF‐β	AA	Phase I	USA; Egypt	NCT03078686 NCT05251831
	Amlitelimab	OX40/OX40L inhibitor	OX40L	Severe AA in adults	Phase II	USA	NCT06444451
	IMG‐007		OX40	AA in adults	Phase II	USA	NCT06060977
Small molecule drug	Tofacitinib	JAK inhibitor	JAK1/3	Extensive and persistent AA	Phase IV	Thailand	NCT03800979
	Ifedanzitinib (ATI‐502)			AA, AU, and AT	Terminate	USA	NCT03759340
	ATI‐501			AA, AU, and AT in adults	Phase II	USA	NCT03594227
	Ruxolitinib		JAK1/2	Moderate to severe AA	Phase II	USA	NCT01950780
	Baricitinib			Severe AA in adults	US FDA approval to market	USA	NCT03570749 NCT03899259
	Deuruxolitinib(CTP‐543)			Moderate to severe AA in adults	Phase III	USA	NCT04518995 NCT04797650
	Brepocitinib(PF‐06700841)		JAK1/TYK2	Moderate to severe AA	Phase II	USA	NCT02974868 NCT05076006
	Jaktinib		JAK1/2/3	Severe AA	Phase III	China	NCT05255237
	Ritlecitinib (PF‐06651600)		JAK3/TEC	Severe AA in adults and adolescents 12 years and older	US FDA approval to market	USA	NCT02974868 NCT03732807
	Upadacitinib		JAK1	Severe AA in adults and adolescents	Phase III	USA	NCT06012240
	Ivarmacitinib (SHR0302)		JAK1	Moderate to severe patchy alopecia	Phase III	China	NCT05470413
	SYHX1901		JAK/SYK	Severe AA	Phase II	China	NCT06562894
	Abatacept	T‐cell modulation	CD80/86	Moderate to severe patchy alopecia	Phase II	USA	NCT02018042
	Alefacept		CD2	Chronic, severe scalp AA	Not Applicable	USA	NCT00167102
	Efalizumab		CD11A	AT, AU, and severe AA	Phase II	Not provided	NCT00746980
	Apremilast	Phosphodiesterase inhibitor	PDE‐4	Moderate to severe AA	Not Applicable	USA	NCT02684123
	BNZ‐1	IL‐2/IL‐9/IL‐15 Inhibitor	IL‐2, IL‐9, IL‐15	Moderate to severe AA	Phase II	Not provided	NCT03532958
	EQ101			Moderate to severe AA in adults	Phase II	Australia	NCT05589610
	STS‐01	Cytokines modulator	Proinflammatory	Mild to moderate AA	Phase II	UK	NCT06402630

*Data sources*: ClinicalTrials.gov.

### 4.1 Traditional Treatments

AA is traditionally treated through pharmacological interventions that target the regulation of immune responses and hair growth [177]. Common treatment methods include intralesional corticosteroid injections, topical potent corticosteroids, oral corticosteroids, and local contact immunotherapy, among others. While these treatments can help manage the symptoms of AA to some extent, their effectiveness varies from person to person. Additionally, most of these therapies come with potential side effects and risks, which limits their overall clinical effectiveness. Therefore, while these traditional approaches may offer relief, they are not without their limitations (Table 3).

**TABLE 3:** Traditional treatments for AA.

Classifications	Drugs	Mechanism of action	Styles	Adverse reactions	Disadvantages
Glucocorticosteroid	TNT	Glucocorticoids have anti‐inflammatory, immunosuppressive, and antiallergic pharmacological effects, and they suppress inflammatory and immune responses around the hair follicle, leading to the treatment of AA.	Local injection of trimethoprim [178]	Localized pain, skin atrophy, depigmentation, capillary dilatation, and folliculitis	The relatively low effective rate, high side effects, and tendency to recur after stopping the drug are only recommended for patients with acute AA to avoid rapid progression of AA to AT or AU.
	Compound betamethasone		Local injection of compound betamethasone		
	Clobetasol propionate		Topical application of clobetasol propionate [179]		
	Mometasone		Topical application of mometasone furoate [180]		
	Dexamethasone		Systemic oral dexamethasone [181]	Weight gain, elevated blood pressure and glucose, acne, hirsutism, osteoporosis, peptic ulcers, adrenal suppression, and difficulty in wound healing	
	Prednisolone		Intravenous prednisolone [182]		
	Prednisone		Systemic oral prednisone [183]		
	Methylprednisolone		Intravenous methylprednisolone [184]		
CNI	CsA	CsA can effectively inhibit the proliferation and activation of T cells and inhibit the production of IL‐2 and IFN‐γ, thus blocking the disease progression of AA and exerting immunosuppressive and hair regeneration‐promoting effects.	Systemic oral CsA [185]	Nephrotoxicity, immunosuppression, high blood elevation, hirsutism	Easy to relapse after stopping the drug, ineffective in topical application, prone to nephrotoxicity and neurotoxicity in long‐term use
Folate reductase inhibitor	Methotrexate	After entering the cells, methotrexate can inhibit the proliferation of inflammatory cells and reduce inflammatory factors through different pathways to achieve anti‐inflammatory effects.	Systemic oral or subcutaneous injection of methotrexate [186]	Bone marrow suppression, pancytopenia, rash, acne, mucositis, nausea, diarrhea, hepatotoxicity	It works better only in combination with other medications, tends to recur after discontinuation, has a high rate of recurrence in children with baldness, and may cause persistent nausea.
Antimetabolic immunosuppressant	Azathioprine	Azathioprine, similar to methotrexate, is an immunosuppressant that reduces the proliferation of disease cells and the production of inflammatory factors.	Systemic oral azathioprine [24]	Bone marrow suppression, pancytopenia, rash, nausea, diarrhea, hepatotoxicity, and flu‐like symptoms	It is slow to work, usually requires a combination of medications, and has more side effects.
Vitamin A derivatives	Retinoic acid	The mechanism is unknown, but retinoic acid induces a return to normal cellular differentiation and triggers dermatitis that may contribute to hair regrowth in AA.	Topical retinoic acid [187]	Skin irritation, itching, edema, dermatitis, peeling	Low percutaneous absorption, irritation, may cause fetal malformation, pregnant women or preparing for pregnancy men and women are not recommended to use.
Peripheral vasodilator	Minoxidil	Minoxidil can expand subcutaneous blood vessels, enhance blood circulation, improve the microenvironment around hair follicles, and stimulate hair follicles to proliferate hair growth.	Oral or long‐term topical minoxidil hair gel or foam [188, 189]	Dermatitis, itching (more gel than foam); hair growth in areas not affected by AA	It may result in the loss of stunted hairs, hair loss may become more extensive, and your own constitution may affect the efficacy of the treatment.
Exposure to sensitizer	DPCP and SADBE	Exposure to sensitizers can prime the local immune response, which can stimulate hair growth	Topical use of DPCP and SADBE [190]	Edema, erythema, urticaria, dermatitis, itching, depigmentation, irritation of eyes, and lymphadenopathy	Expensive, unstable nature, less application
Irritant contact agent	Anthralin	Anthralin can induce hair regrowth by generating free radicals leading to irritant contact dermatitis.	Topical anthralin [191]	Dry skin, itching, erythema, skin turning brown, hair and clothes stained brown, even headaches and nausea, vomiting and diarrhea, liver and kidney toxicity	Contact with the eyes may cause severe conjunctivitis, keratitis or corneal clouding, and prolonged use in large quantities may cause systemic symptoms of toxicity.
Phototherapy combined with immunosuppressants	Psoralen photochemotherapy	Psoralens make the skin more sensitive to light, and UV exposure increases blood circulation and stimulates hair follicles, which leads to hair regrowth.	Oral or topical application of psoralen along with ultraviolet UVA irradiation [192]	Edema, erythema, dryness, dermatitis, itching, nausea, and headache	Burning sensation after stopping the drug, may cause skin cancer, high recurrence rate after stopping the drug.

Abbreviations: TNT: triamcinolone acetonide; SADBE: squaric acid dibutyl ester.

Traditional treatments for AA often exhibit unstable efficacy, particularly in cases of extensive or total hair loss, where complete regrowth is unlikely. Even when effective in the short term, these treatments are typically associated with a high relapse rate, which is a key characteristic of the condition. Prolonged use of these therapies can lead to serious side effects, such as localized skin atrophy, folliculitis, and pigmentation changes. Additionally, systemic side effects like osteoporosis and diabetes may also occur. As a result, finding new treatment options and medications, particularly for patients who either do not respond to or cannot tolerate traditional therapies, has become a critical focus of ongoing research.

### 4.2 Biologics

#### 4.2.1 Th17 Inflammatory Signaling Pathway Inhibitors

Secukinumab is a fully human monoclonal antibody, that inhibits the Th17 inflammatory signaling pathway by specifically targeting IL‐17A. It has gained approval for addressing conditions such as plaque psoriasis, arthropathic psoriasis, and ankylosing spondylitis (AS). Other Th17 inhibitors in this category include ixekizumab and brodalumab. Given the pivotal role of IL‐17 in the pathogenesis of AA, Th17 inhibitors are anticipated to be effective against AA [45]. Case studies have demonstrated that following treatment with secukinumab in patients presenting both psoriasis and total AA, there was observable hair regeneration. However, hair loss recurred upon cessation of the medication, underscoring secukinumab's potential in targeting Th17 cytokines for AA treatment [193]. Nevertheless, in a prior randomized clinical trial involving seven AA patients administered subcutaneous secukinumab injections, only one patient exhibited partial hair regrowth, one experienced aggravated hair loss, while the remaining five showed no response [194]. Additionally, there have been reports of psoriasis patients, initially without AA, developing AA subsequent to secukinumab treatment [195]. Despite the theoretical promise of secukinumab in AA treatment, its efficacy remains inconclusive. Furthermore, a Phase II clinical trial aimed at investigating its efficacy in AA treatment (NCT02599129) was terminated due to low enrollment.

#### 4.2.2 Th2 Inflammatory Signaling Pathway Inhibitors

Dupilumab, a fully human monoclonal antibody, functions as a specific blocker of the Th2 inflammatory signaling pathway. By binding to the α‐subunit of the IL‐4 receptor, it effectively blocks downstream signaling of IL‐4 and IL‐13, thereby inhibiting the Th2 inflammatory signaling pathway and corresponding cytokines [196]. Clinically approved for treating patients aged 12 years and older with moderate‐to‐severe atopic dermatitis (AD) and asthma, dupilumab has shown promise in managing these conditions. Several relevant case reports have indicated that in patients with AD complicated by AA, dupilumab demonstrated the ability to alleviate the development of AA while effectively treating AD [197]. However, there have also been reports suggesting that the use of dupilumab to treat AD could induce or exacerbate AA [198, 199, 200]. These seemingly contradictory outcomes underscore the need for further investigation into the exact mechanism underlying these effects. One proposed explanation for the induction or exacerbation of AA during dupilumab treatment of AD is that the drug may induce atrophy of sebaceous glands and nonscarring alopecia. Currently, a phase II clinical trial investigating the efficacy of dupilumab for the treatment of AA (NCT03359356) has been completed. Additionally, a phase II clinical trial focusing on the use of dupilumab for the treatment of pediatric AA (NCT05866562) is anticipated to commence soon. These trials aim to provide more insights into the specific effects of dupilumab on AA.

Tralokinumab, an Ig4 humanized monoclonal antibody, has been given clinical approval to treat moderate‐to‐severe AD in adult patients. This specific blocker of the Th2 inflammatory signaling pathway works by inhibiting the binding of IL‐13 to its receptor and downstream signaling. In a phase II trial of tralokinumab for treating AA (NCT02684097), patients demonstrated a modest improvement in their SALT scores. However, the trial faced challenges as only a limited number of subjects completed it due to the observed lack of efficacy.

#### 4.2.3 IL‐12/IL‐23 Inhibitors

Ustekinumab is a fully humanized monoclonal IgG1 antibody, which inhibits T‐cell development into Th17 and Th1 cells by specifically targeting the p40 component that is shared by IL‐12 and IL‐23. Currently approved for use in moderate‐to‐severe plaque psoriasis, restrictive enterocolitis, and ulcerative colitis, ustekinumab functions as an IL‐12/IL‐23 inhibitor [201]. In a case report, three patients with severe AA treated with ustekinumab exhibited varying degrees of hair regrowth and significant reductions in the levels of inflammatory markers such as Th1, Th2, and PDE4. These markers, previously highly expressed in the patients’ skin biopsies, were notably downregulated. Numerous reports also suggest that ustekinumab can provide relief and promote hair regrowth in AA patients [201, 202]. While there have been no clinical randomized controlled studies to validate the clinical efficacy of ustekinumab in treating AA, the promising results seen in these case reports warrant further exploration of its potential in the future.

Tildrakizumab is a humanized IgG1/k monoclonal antibody that functions as an IL‐23 inhibitor, exerting high‐affinity blockade of the binding between the p19 subunit of IL‐23 and its receptor. This action effectively inhibits the release of proinflammatory cytokines and chemokines. The drug is clinically approved for treating adult patients with moderate to severe plaque psoriasis who require systemic therapy or phototherapy according to the indications. In a prospective study involving nine patients with moderate to severe chronic AA that was refractory to conventional therapy, two patients demonstrated improvement following subcutaneous injection of tildrakizumab [203]. While this study alone does not provide comprehensive data, it suggests the potential effectiveness of tildrakizumab in the treatment of AA.

#### 4.2.4 Small Dose IL‐2

IL‐2, an essential cytokine involved in the regulation of Tregs homeostasis, plays a crucial role in maintaining immune balance. The depletion of Tregs may contribute to the disruption of hair follicular IP, ultimately leading to the development of AA [204]. Studies have indicated that small doses of IL‐2 can effectively stimulate the proliferation of Tregs and restore the Th17/Treg cells balance, thereby inducing and reinstating immune tolerance within hair follicles. This suggests a potential role for IL‐2 in the treatment of AA. A prospective open pilot study demonstrated a significant increase in Tregs numbers at skin lesions in patients with severe AA following subcutaneous injections of low‐dose IL‐2. Concurrently, partial hair regeneration and notable improvement in AA were observed [205]. While multicenter prospective placebo‐controlled studies with larger sample sizes are lacking, there is preliminary evidence supporting the ability of low‐dose IL‐2 to recruit Tregs. Despite these promising findings, further investigation is warranted to fully understand the therapeutic effects and mechanisms of low‐dose IL‐2 in treating AA due to limited available data.

#### 4.2.5 TNF‐α Inhibitors

Patients with autoimmune diseases, particularly those with AA, often exhibit elevated serum TNF‐α levels compared with healthy individuals. Consequently, TNF‐α inhibitors, as primary biologics for managing autoimmune diseases, are naturally anticipated to be effective in treating AA. However, there is a scarcity of comprehensive reports on the use of TNF‐α inhibitors for AA treatment, and the available data mostly consist of small sample sizes. Etanercept, a human TNFR p75 Fc fusion protein produced using a Chinese hamster ovary cell expression system, competes with blood TNF‐α for binding, thereby blocking its interaction with cell surface TNFR and reducing TNF‐α activity. While etanercept has been utilized for treating rheumatoid arthritis (RA), AS, and other immune diseases in several countries, a prospective study involving 20 cases revealed its ineffectiveness in managing moderate to severe AA [206]. Despite enalapril's potential as a treatment for AA based on its targeted mechanism of action, research in this area has become limited due to unsatisfactory data.

Adalimumab, the first recombinant synthetic fully human TNF‐α monoclonal antibody approved for marketing, has gained global approval for treating a range of conditions, including RA, AS, psoriasis, and various other diseases. By specifically binding to soluble human TNF‐α, adalimumab effectively inhibits its interaction with cell surface TNF‐α receptors p55 and p75, thereby blocking the inflammatory effects of TNF‐α and achieving successful treatment outcomes. A notable case report underscores adalimumab's potential in treating AA. The report highlights a female AA patient in her 30s with a history of moderate AD, who, despite undergoing multiple treatments for AA without significant improvement, experienced hair growth within 2 weeks of initiating weekly subcutaneous injections of adalimumab. Remarkably, complete regrowth was observed after 6 months of treatment [207]. While this case provides promising insights, it is essential to acknowledge the limitation of its small sample size. To establish a robust case for the efficacy of adalimumab in treating AA, further experimental studies with larger sample sizes are warranted. These efforts will be crucial in validating the therapeutic potential of adalimumab in managing AA and enhancing patient care.

#### 4.2.6 Platelet‐Rich Plasma

Platelet‐rich plasma (PRP), some biological agent rich in platelets, boasts high concentrations of growth factors and cytokines. Its therapeutic potential lies in promoting tissue repair and hair follicle regeneration by downregulating the expression of MCP‐1 and upregulating TGF‐β, making it a promising treatment for AA [208]. Comparative studies have revealed that PRP therapy surpasses the efficacy of 5% minoxidil and intralesional steroids in treating AA [209, 210]. Additionally, PRP therapy for AA shows a good safety profile, indicating that it may be used as a first‐line treatment in the future. Several clinical studies have been undertaken to explore the efficacy of PRP in treating both AA and AGA, underscoring the growing interest and research in this area.

#### 4.2.7 OX40/OX40L Inhibitors

OX40, also known as CD134 or TNFRSF4, is a costimulatory molecule belonging to the TNFR superfamily. OX40 is expressed predominantly on APS, activated CD4^+^ and CD8^+^ T cells and transiently upon TCRs stimulation [211]. Studies have shown that OX40 is closely associated with a variety of autoimmune diseases, including systemic lupus erythematosus (SLE), RA, inflammatory bowel IBD, and AA [212]. Signaling between OX40 and its ligand, OX40L, enhances Th1‐mediated immune responses, promotes the generation and maintenance of Th2 responses, regulates Th17 cell of IL‐17 production, and inhibit the generation of Tregs and their suppressive effects. In addition, OX40 is essential for CD8^+^ cytotoxic T cells, promoting the survival and proliferation of activated CD8^+^ T cells and enhancing their response to antigenic stimuli [213]. Due to its important role in immunomodulation, OX40 has become a hot research topic in the field of immunotherapy in recent years. However, only a few clinical trials are currently exploring therapeutic strategies targeting OX40 or OX40L.

Amlitelimab, a monoclonal antibody targeting OX40L, is unique in its ability to bind to OX40L, thereby inhibiting T‐cell‐dependent inflammatory responses but not leading to immune cell clearance. In this way amlitelimab can play a role in therapy while avoiding immunosuppression [214]. A study evaluating the efficacy and safety of subcutaneous monotherapy with amlitelimab versus placebo in patients with severe AA is recruiting participants (NCT06444451). The recent news that amlitelimab has also been approved for the treatment of severe AA in China has generated widespread interest.

IMG‐007 is a humanized anti‐OX40 monoclonal antibody that exerts its therapeutic effects by blocking the OX40‐OX40L signaling pathway. Notably, IMG‐007 has a long half‐life of 31 days, which means it may be used to achieve a lower dosing frequency. As the only nondepleting anti‐OX40 monoclonal antibody in clinical phase worldwide, IMG‐007 has an ongoing Phase II clinical trial focused on the treatment of AA (NCT06060977). Overall, the discovery of the OX40/OX40L signaling pathway not only provides important clues for the development of new drug targets, but also offers more possible options for the treatment of diseases such as AA.

### 4.3 Small‐Molecule Drugs

#### 4.3.1 JAK Inhibitors

JAK inhibitor therapy has emerged as a promising and increasingly popular approach in the treatment of AA. This therapy targets the immune system, specifically by addressing CD8^+^ T cells surrounding the hair follicles. By blocking the downstream pathways of key cytokines such as IFN‐γ, IL‐2, IL‐15, and IL‐17, it effectively suppresses T cell production and activation. In addition, it promotes hair follicle cells' development and differentiation, which eventually aids in hair regrowth.

The first‐generation JAK inhibitors, such as tofacitinib, ruxolitinib, and baricitinib, are nonselective and are currently used in clinical settings [215, 216, 217, 218, 219]. These inhibitors have the ability to simultaneously target at least two JAK targets and block multiple related signaling pathways [220, 221]. Specifically, tofacitinib inhibits JAK1/2/3, while ruxolitinib and baricitinib selectively inhibit JAK1/2 [222]. A case report demonstrated nearly complete hair regrowth in skin lesions following oral ruxolitinib treatment in three patients with AA. Biopsies of the skin lesions revealed reduced perifollicular T‐lymphocyte infiltration and decreased expression of HLA class I and class II after treatment [223]. In a study involving 32 Korean patients with severe AA, 75% of the individuals experienced hair regrowth after receiving oral tofacitinib treatment. However, five patients had recurrences after stopping the medication [224]. Similar reports and studies have indicated the efficacy and safety of nonselective JAK inhibitors in treating AA. Notably, baricitinib has obtained United States Fodd and Drug Administration (US FDA) approval as the first systemic therapy for AA and later received approval from the NMPA as the first innovative drug for AA treatment in China. While most nonselective JAK inhibitors have shown promising results in AA case reports and clinical trials, further research is required to establish their suitability for widespread use in AA treatment [220, 223, 225].

Second‐generation JAK inhibitors are a class of selective JAK inhibitors recognized for their enhanced safety profiles and the ability to target specific JAK pathways, thereby inhibiting signaling associated with particular diseases. Notably, the selective JAK3 inhibitor ritlecitinib mesylate stands as the world's first innovative drug approved for treating severe AA in both adolescents and adults aged 12 years and above, presenting a crucial therapeutic option for this patient population. Additionally, the selective JAK1/2 inhibitor deuruxolitinib (CTP‐543), an analog of ruxolitinib, has garnered Breakthrough Therapy Designation and Fast Track status from the US FDA for its exceptional efficacy and safety in treating AA. Several other selective JAK inhibitors for treating AA are currently in various stages of research, development, and filing. Among these, ifedanzitinib (ATI‐502), ATI‐501, brepocitinib (PF‐06700841), jackitinib, upadacitinib, and ivarmacitinib have progressed into the Phase II‐III clinical stage. Currently, JAK inhibitors have been widely used in AD, psoriasis, vitiligo, cutaneous lupus erythematosus, and other diseases. Established studies have shown that both nonselective and selective JAK inhibitors have also shown good potential for the treatment of AA, and are expected to become ideal therapies for AA treatment in the future.

#### 4.3.2 T‐cell Regulations

Abatacept, a soluble CTL‐associated antigen 4 (CTLA‐4), functions as a selective modulator of T cell costimulation. It is a fusion protein comprising CTLA‐4 and the Fc segment of partial IgG1 (CTLA‐4 Ig). By binding to CD80 and CD86 receptors on APC and obstructing the costimulatory effects of the T cell surface molecule CD28, activation of CD8^+^ T cells and the release of inflammatory cytokines linked to AA, namely IL‐2, TNF‐α, and IFN‐γ, are both efficiently inhibited by abatacept [226]. The primary adverse reaction associated with abatacept is upper respiratory tract infection, with no reports of other serious adverse events. In a phase II clinical trial (NCT02018042), 15 patients with moderate to severe AA, treated with weekly subcutaneous injections of abatacept for 6 months, exhibited a mean hair regrowth rate of 21.3% (ranging from 2.9 to 91.0%). Additionally, an open‐label, single‐arm clinical trial demonstrated the therapeutic potential of abatacept in treating AA.

Alefacept is a dimeric protein resulting from the fusion of the extracellular portion of human leukocyte function‐associated antigen‐3 bound to CD2 with the stranded and stabilized (CH2 and CH3) portions of the human immunoglobulin IgG heavy chain. It functions by binding to CD2 molecules on the surface of activated T cells, thereby inhibiting the costimulation of T cells by APC. Additionally, alefacept selectively targets memory effector T cells, leading to their inhibition and depletion of their effects. In a reported case, a 21‐year‐old female patient diagnosed with AU achieved complete regeneration of scalp and body hair after 12 consecutive weeks of once‐weekly treatment with alefacept [227]. Subsequently, a multicenter, double‐blind, randomized, placebo‐controlled clinical trial enrolled 45 patients with chronic, severe AA who had previously received ineffective treatments. Of these, 23 patients received the same treatment regimen described above, while the remaining patients were administered a placebo. However, a comparison of efficacy between the two groups did not reveal a statistically significant difference [228]. The available clinical data are insufficient to conclusively demonstrate that alefacept can effectively cure AA. Further studies are warranted to validate its efficacy in treating this condition.

Efalizumab is a monoclonal antibody preparation targeting CD11a, which works to suppress the immune effects of CD11a by specifically recognizing the CD11a antigen on white blood cells and reducing their ability to adhere to other cells. In a case report, a 19‐year‐old patient with a 4‐year history of AA, experiencing hair loss across the entire body except for the eyebrows and eyelashes, began regrowing hair after just 1 month of efalizumab treatment. As treatment progressed, hair growth extended to other body parts, with no significant side effects, resulting in substantial hair regrowth within 7 months of treatment [229]. This case demonstrates the efficacy of efalizumab in treating AA. However, despite its effectiveness, the use of efalizumab has been associated with the risk of progressive multifocal leukoencephalopathy. Due to this risk, the European Medicines Agency proposed the suspension of efalizumab sales in the European Union in February 2009.

#### 4.3.3 Phosphodiesterase Inhibitors

Apremilast, an oral PDE4 inhibitor, has gained approval for treating active arthropathic psoriasis and moderate to severe plaque psoriasis. Its efficacy in addressing AA has been documented in clinical reports. Notably, a retrospective study showcased significant hair regrowth (over 50%) in 13 out of 15 patients with persistent AA, who had not responded to conventional therapies. These patients experienced positive outcomes after being treated with oral apremilast [230]. Furthermore, studies using a humanized mouse model of AA have demonstrated the effectiveness of apremilast [231]. The principal mechanism of action of apremilast is the elevation of intracellular cyclic adenosine monophosphate (cAMP) levels. Through this method, the expression of IL‐10 is enhanced while that of other inflammatory cytokines, including IFN‐γ, TNF‐α, IL‐12, IL‐17, IL‐22, and IL‐23, is reduced. These actions collectively contribute to diminishing the inflammatory response and safeguarding the normal structure of the hair follicle. Despite the positive results observed in some clinical studies regarding its efficacy in treating AA, further clinical trials are necessary to validate its effectiveness.

#### 4.3.4 IL‐2/IL‐9/IL‐15 Inhibitors

BNZ‐1 is an innovative small molecule targeted drug comprising a PEG peptide molecule designed to selectively inhibit the cytokines IL‐2, IL‐9, and IL‐15. These cytokines play pivotal roles in activating cytotoxic T‐cells and NK cells, contributing to the immunological pathogenesis of AA. By targeting these cytokines, BNZ‐1 shows promise in blocking the onset of AA. Currently, BNZ‐1 is undergoing evaluation in a multicenter randomized double‐blind controlled phase II clinical trial (NCT03532958). This trial represents a significant step toward assessing the efficacy and safety of BNZ‐1 in treating AA.

EQ101 is a novel peptide that combines the high selectivity of monoclonal antibody drugs with the cytokine inhibitory ability of small‐molecule drugs. As a multicytokine inhibitor, EQ101 is able to target and inhibit cytokines IL‐2, IL‐9, and IL‐15, which are key drivers in the pathogenesis of AA, and activate the JAK–STAT signaling pathway. Therefore, EQ101 is expected to be a new option for the treatment of AA. A phase II clinical trial of EQ101 in the treatment of moderate to severe AA in adults has been completed (NCT05589610), which was designed to evaluate its safety and efficacy. Moreover, the concept of simultaneously inhibiting multiple cytokines associated with the immunologic pathogenesis of AA suggests a novel direction in the treatment of this condition.

#### 4.3.5 Cytokines Modulators

STS‐01 is an innovative small molecule cytokine modulator that exerts therapeutic effects by modulating the inflammatory response and proliferation of T cells and interfering with related signaling pathways. Its mechanism is based on a well‐defined safety profile with good potential. Recently announced results from a Phase II clinical trial of STS‐01 in the treatment of patients with mild to moderate AA showed positive results (NCT06402630). The trial involved a total of 158 participants randomly assigned to different concentrations of STS‐01 treatment groups (0.25, 0.5, 1, or 2%) and a placebo group for a 24‐week treatment period. Efficacy was assessed primarily by improvement in SALT scores. Clinical results showed that patients treated with STS‐01 exhibited significant hair regrowth over the course of treatment. In particular, 19 and 27% of patients in the 1 and 2% concentration groups achieved 100% scalp hair coverage, respectively, while only 3% of patients in the placebo group reached this result. In addition, STS‐01 demonstrated good tolerability in clinical trials with no significant adverse events. These data demonstrate the safety and efficacy of STS‐01 in the treatment of mild to moderate AA, suggesting that it is expected to become a standard treatment option for AA. The study also provides new therapeutic ideas for treating AA by intervening in specific responses of the immune system.

### 4.4 Other Therapies

#### 4.4.1 Combination Drug Therapy

In clinical practice, the strategic use of drug combinations is often employed to optimize treatment outcomes for patients. This approach aims to deliver the most effective therapeutic results in the shortest possible time while mitigating the toxic side effects and development of drug resistance. By combining drugs, it is possible to alleviate patients' discomfort and enhance treatment efficacy. The ideal drug combination can effectively reduce toxicity and adverse reactions, create synergistic effects among drugs to amplify therapeutic benefits, and mitigate the onset of drug resistance. Furthermore, it facilitates the rapid, comprehensive eradication of pathogenic bacteria, thus lessening the economic burden on patients. Given these numerous advantages, the treatment of AA also frequently involves the use of drug combinations.

The combination of methotrexate and low‐dose prednisone has emerged as a promising treatment for AT and AU, offering improved efficacy and tolerability compared with methotrexate alone. This regimen is also recognized as a more cost‐effective option for treating AA [232]. Clinical evidence suggests that while methotrexate monotherapy may not be sufficiently effective for AT and AU, combining it with low‐dose prednisone can lead to near‐complete hair regrowth in up to 31% of patients. Importantly, this combination therapy is associated with a reduced risk of hormone‐related adverse effects, enhancing its safety profile.

Latanoprost, when combined with betamethasone and minoxidil, emerges as a promising adjunctive treatment option [233]. A study involving 108 patients of both sexes with AA showcased the efficacy of topical latanoprost in enhancing the effectiveness of topical betamethasone and minoxidil, without inducing any adverse effects. This combination therapy demonstrated safety and effectiveness in treating patchy alopecia.

#### 4.4.2 Microneedle‐Based Therapy

Microneedles (MN) represent a pioneering physically facilitated penetration technique, comprising numerous micron‐sized fine tips arranged in an array on a base. This innovation has found diverse applications in dermatology, particularly in the treatment of various dermatologic conditions. By creating numerous microscopic pore channels in the stratum corneum of the skin, MN enhance the penetration of topical medications, facilitating the delivery of drugs that are not easily absorbed by the skin and significantly improving their absorption and utilization [234]. Additionally, it has been shown that MN supports epidermal regeneration and repair. They achieve this by upregulating the Wnt/β‐catenin pathway, stimulating the production of multiple growth factors, and inducing capillary formation through microinjuries in the epidermis [235, 236]. An additional benefit is the reduction in patient discomfort, as the use of MN instead of traditional needles improves the overall treatment experience. In the context of AA, MN has shown potential in enhancing blood supply to the hair follicles by stimulating the dermal papillae and hair follicle stem cells, thereby promoting hair growth [237, 238]. While MN in combination with tretinoin, compound betamethasone, and minoxidil has shown promise, it is worth noting that current research on MN for hair loss primarily focuses on AGA, with fewer studies examining its efficacy in treating AA [239, 240, 241].

#### 4.4.3 Exosomes

Extracellular vesicles (EVs) are tiny vesicles secreted by cells, typically ranging in diameter from 30 to 150 nm. They are present in various biological fluids such as blood, saliva, and lymph, and are rich in bioactive substances with therapeutic potential [242, 243]. Exosomes, a subtype of EVs, possess the unique ability to traverse cell membranes and are less likely to trigger immune responses, making them increasingly utilized in in vivo therapeutic applications and as carriers for drug delivery [244, 245]. Numerous clinical trials focusing on wound healing, melanoma, AD, SLE, psoriasis, hair regrowth, and other dermatological conditions have underscored their therapeutic promise [246, 247, 248]. Stem cell‐derived exosomes, in particular, have garnered significant attention for their role in repairing damaged hair follicles and promoting hair regeneration. Recent studies have demonstrated that mesenchymal stem cell‐derived exosomes can activate DPCs, enhancing their proliferation and migration abilities in mice. Growth factors including IGF‐1 and VEGF are secreted as a result of this stimulation, which eventually promotes hair regeneration [249]. Moreover, research has revealed that exosomes derived from dermal cells with high expression of miR‐218‐5p can stimulate hair growth by modulating β‐catenin signaling and transitioning hair follicles from the resting to the growth phase [250]. Additionally, efforts have been made to combine exosomes with MN for hair growth stimulation. For instance, MN comprising polyvinyl alcohol tips and hyaluronic acid (HA) substrates loaded with chitosan lactate and adipose‐derived stem cell exosomes have shown promising therapeutic outcomes [251]. The expanding body of evidence underscores the significant role of exosomes in the treatment of AA, hinting at their potential as an effective therapeutic modality in the future.

#### 4.4.4 Nanotechnology

Nanoparticles (NPs) are microscopic particles artificially manufactured with sizes ranging from 1–100 nanometers. Due to their unique structure, NPs typically possess a large specific surface area and exhibit high biocompatibility and biodegradability [252]. In recent years, NPs have found increasing applications in healthcare, cosmetic research and development, and biomaterials. Particularly in medicine, NPs offer numerous advantages such as enhancing drug stability, solubility, and concentration, as well as prolonging drug release while minimizing adverse effects on cellular growth and metabolism [253]. Notably, NPs not only exhibit a tendency to target hair follicle cells but also aggregate around them, making nanotechnology a promising approach for treating follicle‐related disorders like AA [254]. Studies have demonstrated that certain metal‐based NPs exhibit excellent follicular permeability, penetrating deeply into follicular pores for sustained retention within the follicles [255]. In addition, NPs encapsulating minoxidil, prepared through grafting HA with poly (lactic‐co‐glycolic acid) (HA‐PLGA), have been shown effective in targeting and safely interacting with hair follicle cells in various experimental assays, including cell viability, cellular uptake, and skin penetration tests [256]. Further research involved the development of NP gels using PLGA for delivering tofacitinib citrate, which significantly enhanced drug penetration and activity [257]. In conclusion, nanotechnology holds promise as a safe and effective method for treating AA, leveraging its unique properties to optimize drug delivery and therapeutic outcomes.

## 5 Conclusion and Future Directions

AA is a prevalent autoimmune condition marked by the sudden and localized loss of hair. Although significant progress has been made in understanding its u pathogenesis and exploring treatment options, several aspects of AA remain poorly understood, leading to substantial gaps in its effective management. This section aims to highlight the future directions and ongoing challenges in AA research, with a particular focus on its pathogenesis, diagnostic approaches, and treatment strategies.

While it is understood that the onset of AA is linked to immune‐mediated damage to hair follicles, the precise immunological mechanisms underlying its pathogenesis remain elusive. Immune‐mediated mechanisms, environmental and epigenetic factors, hair follicle cycle disruption, and genetic predisposition are all believed to play a role, but further rigorous research is needed to clarify their involvement in disease onset and progression. A deeper and more comprehensive understanding of AA's pathogenesis could lead to more effective clinical treatments, facilitate the development of personalized medicine, and ultimately enable early diagnosis, precise therapies, and better prevention strategies. This would have significant social and medical implications, enhancing patient outcomes and reducing the overall disease burden.

Current data highlight a significant lack of drugs targeting AA in both development and clinical use. At present, there is no definitive cure for AA. Common treatment options include the topical or systemic use of glucocorticoids, minoxidil, and light therapy. However, these therapies come with limitations, such as modest efficacy, notable side effects, and a tendency for recurrence [19, 26]. In recent years, there has been a growing interest in clinical trials investigating small molecule targeted drugs and biologic agents for skin diseases. These novel therapies have garnered attention due to their promising efficacy and safety profiles. Of particular note, the development of immunotherapeutic drugs like JAK inhibitors has shown encouraging results in treating AA. While these targeted therapies offer renewed hope for patients, it is important to recognize that much of the supporting evidence comes from small‐scale clinical trials and case reports. Large‐scale trials are needed to more definitively assess the reliability and safety of these treatments. Moreover, the number of drugs currently in development for AA is limited, with only a handful of options available worldwide, primarily focused on immunosuppression and immune regulation. Additionally, while research has identified key targets and pathways implicated in AA's pathogenesis, which hold promise as therapeutic targets, these findings still face challenges. Most studies have been conducted in animal models or organoid systems, which may not fully replicate the complexities of human disease. Consequently, although these targets and pathways show considerable theoretical potential, their clinical effectiveness and feasibility in human treatments require further validation. AA remains a disease that presents substantial challenges, particularly in moderate to severe cases.

It is encouraging to note that, a number of new drugs are under development globally against AA, involving a number of innovative targets such as OX40, ILT7, DHODH, TP53, S1P, and others. These drugs are currently in various stages of preclinical studies, clinical phase I, clinical phase II, and clinical application for approval, demonstrating strong R&D momentum. In addition to the existing treatments, there is growing interest in emerging technologies, such as regenerative medicine techniques (e.g., stem cell therapy and hair follicle bioengineering) and artificial intelligence (AI)‐driven drug design (e.g., nanoenzymes). Although these approaches are still in the experimental phase, they hold great potential for the future. As scientific advancements and societal progress continue, these innovations are expected to offer promising solutions, particularly for patients with severe or treatment‐resistant AA.

Early diagnosis of AA remains a significant challenge, primarily due to its reliance on clinical presentation and the exclusion of other causes of hair loss. The lack of a definitive biomarker for AA further complicates both diagnosis and disease monitoring. Although scalp biopsies and dermoscopy are valuable diagnostic tools, they are invasive and not routinely employed in clinical practice. Therefore, there is an urgent need for the development of noninvasive diagnostic methods, such as serum biomarkers and advanced imaging techniques, capable of detecting AA in its earliest stages. Recent advancements in gene expression profiling and proteomics show promise in identifying potential biomarkers for AA. These biomarkers could facilitate earlier diagnosis, improve predictions of disease progression, and enable more personalized treatment approaches. Furthermore, the integration of AI‐based diagnostic tools, including machine learning algorithms for analyzing dermoscopic images, could significantly enhance the accuracy and efficiency of AA diagnosis. This represents a promising direction for the future of AA diagnostics.

In conclusion, while the understanding of AA has advanced significantly in recent years, much remains to be done to fully unravel the complex pathogenesis of this disease. The development of novel diagnostic tools and targeted therapeutic strategies offers hope for more effective management of AA in the future. However, there are still critical gaps in our knowledge, particularly regarding the triggers of the autoimmune response, the long‐term safety of new therapies, and the need for personalized approaches based on genetic and environmental factors. Continued research in these areas will be essential to improving the lives of individuals affected by AA, providing them with more effective, safer, and personalized treatment options.

## Author Contributions

Tianyou Ma: conceptualization, methodology, and writing—original draft preparation. Tingrui Zhang: conceptualization and writing—reviewing and editing. Fengze Miao: methodology and software. Jun Liu: software and investigation. Quangang Zhu: investigation and visualization. Zhongjian Chen: supervision and resources. Zongguang Tai: investigation, and writing—reviewing and editing. Zhigao He: validation and writing—reviewing and editing. All authors approved the final manuscript.

## Conflicts of Interest

The authors declare no conflicts of interest.

## Ethics Statement

The authors have nothing to report.

## Data Availability

The authors have nothing to report.

## References

[1] T. Simakou , J. P. Butcher , S. Reid , and F. L. Henriquez , “Alopecia Areata: A Multifactorial Autoimmune Condition,” Journal of Autoimmunity 98 (2019): 74–85. 30558963 10.1016/j.jaut.2018.12.001

[2] H. H. Lee , E. Gwillim , K. R. Patel , et al., “Epidemiology of Alopecia Areata, Ophiasis, Totalis, and Universalis: A Systematic Review and Meta‐Analysis,” Journal of the American Academy of Dermatology 82, no. 3 (2020): 675–682. 31437543 10.1016/j.jaad.2019.08.032

[3] A. Nanda , A. S. Al‐Fouzan , and F. Al‐Hasawi , “Alopecia Areata in Children: A Clinical Profile,” Pediatric Dermatology 19, no. 6 (2002): 482–485. 12437546 10.1046/j.1525-1470.2002.00215.x

[4] L. C. Strazzulla , E. H. C. Wang , L. Avila , et al., “Alopecia Areata: Disease Characteristics, Clinical Evaluation, and New Perspectives on Pathogenesis,” Journal of the American Academy of Dermatology 78, no. 1 (2018): 1–12. 29241771 10.1016/j.jaad.2017.04.1141

[5] A. Toussi , V. R. Barton , S. T. Le , O. N. Agbai , and M. Kiuru , “Psychosocial and Psychiatric Comorbidities and Health‐Related Quality of Life in Alopecia Areata: A Systematic Review,” Journal of the American Academy of Dermatology 85, no. 1 (2021): 162–175. 32561373 10.1016/j.jaad.2020.06.047 PMC8260215

[6] P. M. Russo , E. Fino , C. Mancini , M. Mazzetti , M. Starace , and B. M. Piraccini , “HrQoL in Hair Loss‐Affected Patients With Alopecia Areata, Androgenetic Alopecia and Telogen Effluvium: The Rrole of Personality Traits and Psychosocial Anxiety,” Journal of the European Academy of Dermatology and Venereology 33, no. 3 (2019): 608–611. 30394586 10.1111/jdv.15327

[7] S. Aghaei , N. Saki , E. Daneshmand , and B. Kardeh , “Prevalence of pPsychological Disorders in Patients With Alopecia Areata in Comparison With Normal Subjects,” ISRN Dermatology 2014 (2014): 304370. 24734190 10.1155/2014/304370 PMC3966411

[8] T. P. Joshi , D. Garcia , F. Gedeon , et al., “Epidemiology of Alopecia Areata in the Hispanic/Latinx Community: A Cross‐Sectional Analysis of the All of Us Database,” Journal of the American Academy of Dermatology 89, no. 1 (2023): e61–e62. 36921806 10.1016/j.jaad.2023.02.054

[9] S. Ly , P. Manjaly , K. Kamal , et al., “Comorbid Conditions Associated With Alopecia Areata: A Systematic Review and Meta‐Analysis,” American Journal of Clinical Dermatology 24, no. 6 (2023): 875–893. 37464249 10.1007/s40257-023-00805-4

[10] S. Lee , H. Lee , C. H. Lee , and W. S. Lee , “Comorbidities in Alopecia Areata: A Systematic Review and Meta‐Analysis,” Journal of the American Academy of Dermatology 80, no. 2 (2019): 466–477. e16. 30031145 10.1016/j.jaad.2018.07.013

[11] L. Y. Liu , B. A. King , and B. G. Craiglow , “Health‐Related Quality of Life (HRQoL) among Patients With Alopecia Areata (AA): A Systematic Review,” Journal of the American Academy of Dermatology 75, no. 4 (2016): 806–812. e3. 27436156 10.1016/j.jaad.2016.04.035

[12] T. Cartwright , N. Endean , and A. Porter , “Illness Perceptions, Coping and Quality of Life in Patients With Alopecia,” British Journal of Dermatology 160, no. 5 (2009): 1034–1039. 19183424 10.1111/j.1365-2133.2008.09014.x

[13] A. F. Alexis , R. Dudda‐Subramanya , and A. A. Sinha , “Alopecia Areata: Autoimmune Basis of Hair Loss,” European Journal of Dermatology 14, no. 6 (2004): 364–370. 15564197

[14] A. Gilhar , R. Laufer‐Britva , A. Keren , and R. Paus , “Frontiers in Alopecia Areata Pathobiology Research,” Journal of Allergy and Clinical Immunology 144, no. 6 (2019): 1478–1489. 31606262 10.1016/j.jaci.2019.08.035

[15] J. Y. Niederkorn , “See no Evil, Hear no Evil, Do no Evil: The Lessons of Immune Privilege,” Nature Immunology 7, no. 4 (2006): 354–359. 16550198 10.1038/ni1328

[16] F. Rajabi , L. A. Drake , M. M. Senna , and N. Rezaei , “Alopecia Areata: A Review of Disease Pathogenesis,” British Journal of Dermatology 179, no. 5 (2018): 1033–1048. 29791718 10.1111/bjd.16808

[17] M. Bertolini , K. McElwee , A. Gilhar , S. Bulfone‐Paus , and R. Paus , “Hair Follicle Immune Privilege and Its Collapse in Alopecia Areata,” Experimental Dermatology 29, no. 8 (2020): 703–725. 32682334 10.1111/exd.14155

[18] A. G. Messenger , J. McKillop , P. Farrant , A. J. McDonagh , and M. Sladden , “British Association of Dermatologists' Guidelines for the Management of Alopecia Areata 2012,” British Journal of Dermatology 166, no. 5 (2012): 916–926. 22524397 10.1111/j.1365-2133.2012.10955.x

[19] L. C. Strazzulla , E. H. C. Wang , L. Avila , et al., “Alopecia Areata: An Appraisal of New Treatment Approaches and Overview of Current Therapies,” Journal of the American Academy of Dermatology 78, no. 1 (2018): 15–24. 29241773 10.1016/j.jaad.2017.04.1142

[20] B. E. Yee , Y. Tong , A. Goldenberg , and T. Hata , “Efficacy of Different Concentrations of Intralesional Triamcinolone Acetonide for Alopecia Areata: A Systematic Review and Meta‐Analysis,” Journal of the American Academy of Dermatology 82, no. 4 (2020): 1018–1021. 31843657 10.1016/j.jaad.2019.11.066

[21] S. Lee , B. J. Kim , Y. B. Lee , and W. S. Lee , “Hair Regrowth Outcomes of Contact Immunotherapy for Patients With Alopecia Areata: A Systematic Review and Meta‐Analysis,” ISRN Dermatology 154, no. 10 (2018): 1145–1151. 10.1001/jamadermatol.2018.2312 PMC6233743 30073292

[22] B.J Kim, S. Lee, C.H. Lee, W.S. Lee, “Home‐Based Contact Immunotherapy With Diphenylcyclopropenone Improves Compliance With the Recommended Follow‐Up for Patients With Alopecia Areata: A Retrospective Cohort Study,” Journal of the American Academy of Dermatology 82, no. 5 (2020): 1223–1225. 31678469 10.1016/j.jaad.2019.10.043

[23] K. Phan , V. Ramachandran , and D. F. Sebaratnam , “Methotrexate for Alopecia Areata: A Systematic Review and Meta‐Analysis,” Journal of the American Academy of Dermatology 80, no. 1 (2019): 120–127. 30003990 10.1016/j.jaad.2018.06.064

[24] S. Vañó‐Galván , Á. Hermosa‐Gelbard , N. Sánchez‐Neila , et al., “Treatment of Recalcitrant Ddult Alopecia Areata Universalis With Oral Azathioprine,” Journal of the American Academy of Dermatology 74, no. 5 (2016): 1007–1008. 27085230 10.1016/j.jaad.2015.12.055

[25] M. Ohyama , A. Shimizu , K. Tanaka , and M. Amagai , “Experimental Evaluation of Ebastine, a Second‐Generation Anti‐Histamine, as a Supportive Medication for Alopecia Areata,” Journal of Dermatological Science 58, no. 2 (2010): 154–157. 20388588 10.1016/j.jdermsci.2010.03.009

[26] S. Mlacker , A. S. Aldahan , B. J. Simmons , et al., “A Review on Laser and Light‐Based Therapies for Alopecia Areata,” Journal of Cosmetic and Laser Therapy 19, no. 2 (2017): 93–99. 27802065 10.1080/14764172.2016.1248440

[27] A. Alkhalifah , A. Alsantali , E. Wang , K. J. McElwee , and J. Shapiro , “Alopecia Areata Update: Part I. Clinical Picture, Histopathology, and Pathogenesis,” Journal of the American Academy of Dermatology 62, no. 2 (2010): 177–188. quiz 189–90. 20115945 10.1016/j.jaad.2009.10.032

[28] L. Xing , Z. Dai , A. Jabbari , et al., “Alopecia Areata is Driven by Cytotoxic T Lymphocytes and is Reversed by JAK Inhibition,” Nature Medicine 20, no. 9 (2014): 1043–1049. 10.1038/nm.3645 PMC4362521 25129481

[29] A. K. Gupta , T. Wang , S. Polla Ravi , M. A. Bamimore , V. Piguet , and A. Tosti , “Systematic Review of Newer Agents for the Management of Alopecia Areata in Adults: Janus Kinase Inhibitors, Biologics and Phosphodiesterase‐4 Inhibitors,” Journal of the European Academy of Dermatology and Venereology 37, no. 4 (2023): 666–679. 36478475 10.1111/jdv.18810

[30] N. Zhang and M. J. Bevan , “CD8(+) T Cells: Foot Soldiers of the Immune System,” Immunity 35, no. 2 (2011): 161–168. 21867926 10.1016/j.immuni.2011.07.010 PMC3303224

[31] M. Reina‐Campos , N. E. Scharping , and A. W. Goldrath , “CD8(+) T Cell Metabolism in Infection and Cancer,” Nature Reviews Immunology 21, no. 11 (2021): 718–738. 10.1038/s41577-021-00537-8 PMC8806153 33981085

[32] T. Ito , N. Ito , M. Saatoff , et al., “Maintenance of Hair Follicle Immune Privilege is Linked to Prevention of NK Cell Attack,” Journal of Investigative Dermatology 128, no. 5 (2008): 1196–1206. 18160967 10.1038/sj.jid.5701183

[33] A. Gilhar , A. G. Schrum , A. Etzioni , H. Waldmann , and R. Paus , “Alopecia Areata: Animal Models Illuminate Autoimmune Pathogenesis and Novel Immunotherapeutic Strategies,” Autoimmunity Reviews 15, no. 7 (2016): 726–735. 26971464 10.1016/j.autrev.2016.03.008 PMC5365233

[34] J. Zhu , H. Yamane , and W. E. Paul , “Differentiation of Effector CD4 T Cell Populations (*),” Annual Review of Immunology 28 (2010): 445–489. 10.1146/annurev-immunol-030409-101212 PMC3502616 20192806

[35] J. Borst , T. Ahrends , N. Bąbała , C. J. M. Melief , and W. Kastenmüller , “CD4(+) T Cell Help in Cancer Immunology and Immunotherapy,” Nature Reviews Immunology 18, no. 10 (2018): 635–647. 10.1038/s41577-018-0044-0 30057419

[36] R. V. Luckheeram , R. Zhou , A. D. Verma , and B. Xia , “CD4⁺T Cells: Differentiation and Functions,” Clinical & Developmental Immunology 2012 (2012): 925135. 22474485 10.1155/2012/925135 PMC3312336

[37] J. Zhu and W. E. Paul , “CD4 T Cells: Fates, Functions, and Faults,” Blood 112, no. 5 (2008): 1557–1569. 18725574 10.1182/blood-2008-05-078154 PMC2518872

[38] N. Todes‐Taylor , R. Turner , G. S. Wood , P. T. Stratte , and V. B. Morhenn , “T Cell Subpopulations in Alopecia Areata,” Journal of the American Academy of Dermatology 11, no. 2 Pt 1 (1984): 216–223. 6384283 10.1016/s0190-9622(84)70152-6

[39] K. J. McElwee , P. Freyschmidt‐Paul , R. Hoffmann , et al., “Transfer of CD8(+) Cells Induces Localized Hair Loss Whereas CD4(+)/CD25(‐) Cells Promote Systemic Alopecia Areata and CD4(+)/CD25(+) Cells Blockade Disease Onset in the C3H/HeJ Mouse Model,” Journal of Investigative Dermatology 124, no. 5 (2005): 947–957. 15854035 10.1111/j.0022-202X.2005.23692.x

[40] H. J. Michie , C. A. Jahoda , R. F. Oliver , and B. E. Johnson , “The DEBR Rat: An Animal Model of Human Alopecia Areata,” British Journal of Dermatology 125, no. 2 (1991): 94–100. 1911310 10.1111/j.1365-2133.1991.tb06054.x

[41] E. S. Jerud , G. Bricard , and S. A. Porcelli , “CD1d‐Restricted Natural Killer T Cells: Roles in Tumor Immunosurveillance and Tolerance,” Transfusion Medicine and Hemotherapy 33, no. 1 (2006): 18–36.

[42] A. Ghraieb , A. Keren , A. Ginzburg , et al., “INKT Cells Ameliorate Human Autoimmunity: Lessons From Alopecia Areata,” Journal of Autoimmunity 91 (2018): 61–72. 29680372 10.1016/j.jaut.2018.04.001

[43] M. Ono , “Control of Regulatory T‐Cell Differentiation and Function by T‐Cell Receptor Signalling and Foxp3 Transcription Factor Complexes,” Immunology 160, no. 1 (2020): 24–37. 32022254 10.1111/imm.13178 PMC7160660

[44] X. Zhang , N. Olsen , and S. G. Zheng , “The Progress and Prospect of Regulatory T Cells in Autoimmune Diseases,” Journal of Autoimmunity 111 (2020): 102461. 32305296 10.1016/j.jaut.2020.102461

[45] S. H. Loh , H. N. Moon , B. L. Lew , and W. Y. Sim , “Role of T Helper 17 Cells and T Regulatory Cells in Alopecia Areata: Comparison of Lesion and Serum Cytokine between Controls and Patients,” Journal of the European Academy of Dermatology and Venereology 32, no. 6 (2018): 1028–1033. 29283462 10.1111/jdv.14775

[46] S. N. Mueller and L. K. Mackay , “Tissue‐Resident Memory T cells: Local Specialists in Immune Defence,” Nature Reviews Immunology 16, no. 2 (2016): 79–89. 10.1038/nri.2015.3 26688350

[47] H. Xu , R. Zhou , and Z. Chen , “Tissue‐Resident Memory T Cell: Ontogenetic Cellular Mechanism and Clinical Translation,” Clinical and Experimental Immunology 214, no. 3 (2023): 249–259. 37586053 10.1093/cei/uxad090 PMC10719502

[48] S. C. Sasson , C. L. Gordon , S. N. Christo , P. Klenerman , and L. K. Mackay , “Local Heroes or Villains: Tissue‐Resident Memory T cells in Human Health and Disease,” Cellular & Molecular Immunology 17, no. 2 (2020): 113–122. 31969685 10.1038/s41423-019-0359-1 PMC7000672

[49] G. E. Ryan , J. E. Harris , and J. M. Richmond , “Resident Memory T Cells in Autoimmune Skin Diseases,” Frontiers in Immunology 12 (2021): 652191. 34012438 10.3389/fimmu.2021.652191 PMC8128248

[50] M. Lawand , J. Déchanet‐Merville , and M. C. Dieu‐Nosjean , “Key Features of Gamma‐Delta T‐Cell Subsets in Human Diseases and Their Immunotherapeutic Implications,” Frontiers in Immunology 8 (2017): 761. 28713381 10.3389/fimmu.2017.00761 PMC5491929

[51] Y. Uchida , J. Gherardini , A. Schulte‐Mecklenbeck , et al., “Pro‐Inflammatory Vδ1(+)T‐Cells Infiltrates Are Present in and around the Hair Bulbs of Non‐Lesional and Lesional Alopecia Areata Hair Follicles,” Journal of Dermatological Science 100, no. 2 (2020): 129–138. 33039243 10.1016/j.jdermsci.2020.09.001

[52] R. Paus , S. Bulfone‐Paus , and M. Bertolini , “Hair Follicle Immune Privilege Revisited: The Key to Alopecia Areata Management,” Journal of Investigative Dermatology Symposium Proceedings 19, no. 1 (2018): S12–S17. 29273098 10.1016/j.jisp.2017.10.014

[53] E. D. Cetin , E. Savk , M. Uslu , M. Eskin , and A. Karul , “Investigation of the Inflammatory Mechanisms in Alopecia Areata,” American Journal of Dermatopathology 31, no. 1 (2009): 53–60. 19155726 10.1097/DAD.0b013e318185a66e

[54] X. Zhang , Y. Zhao , Y. Ye , et al., “Lesional Infiltration of Mast Cells, Langerhans Cells, T Cells and Local Cytokine Profiles in Alopecia Areata,” Archives of Dermatological Research 307, no. 4 (2015)L 319–331. 25638328 10.1007/s00403-015-1539-1

[55] M. El Darouti , S. A. Marzouk , and E. Sharawi , “Eosinophils in Fibrous Tracts and Near Hair Bulbs: A Helpful Diagnostic Feature of Alopecia Areata,” Journal of the American Academy of Dermatology 42, no. 2 Pt 1 (2000): 305–307. 10.1016/s0190-9622(00)90152-x 10642698

[56] Y. Zhao , B. Zhang , S. Caulloo , X. Chen , Y. Li , and X. Zhang , “Diffuse Alopecia Areata is Associated With Intense Inflammatory Infiltration and CD8+ T Cells in Hair Loss Regions and an Increase in Serum IgE Level,” Indian Journal of Dermatology, Venereology and Leprology 78, no. 6 (2012): 709–714. 23075639 10.4103/0378-6323.102361

[57] A. G. Messenger , D. N. Slater , and S. S. Bleehen , “Alopecia Areata: Alterations in the Hair Growth Cycle and Correlation With the Follicular Pathology,” British Journal of Dermatology 114, no. 3 (1986): 337–347. 3954954 10.1111/j.1365-2133.1986.tb02825.x

[58] T. Kasahara , J. J. Hooks , S. F. Dougherty , and J. J. Oppenheim , “Interleukin 2‐Mediated Immune Interferon (IFN‐Gamma) Production by Human T Cells and T Cell Subsets,” Journal of Immunology 130, no. 4 (1983): 1784–1789. 6403613

[59] F. Castro , A. P. Cardoso , R. M. Gonçalves , K. Serre , and M. J. Oliveira , “Interferon‐Gamma at the Crossroads of Tumor Immune Surveillance or Evasion,” Frontiers in Immunology 9 (2018): 847. 29780381 10.3389/fimmu.2018.00847 PMC5945880

[60] X. Hu and L. B. Ivashkiv , “Cross‐Regulation of Signaling Pathways by Interferon‐Gamma: Implications for Immune Responses and Autoimmune Diseases,” Immunity 31, no. 4 (2009): 539–550. 19833085 10.1016/j.immuni.2009.09.002 PMC2774226

[61] A. Gilhar , Y. Kam , B. Assy , and R. S. Kalish , “Alopecia Areata Induced in C3H/HeJ Mice by Interferon‐Gamma: Evidence for Loss of Immune Privilege,” Journal of Investigative Dermatology 124, no. 1 (2005): 288–289. 15654992 10.1111/j.0022-202X.2004.23580.x

[62] P. Freyschmidt‐Paul , K. J. McElwee , R. Hoffmann , et al., “Interferon‐Gamma‐Deficient Mice are Resistant to the Development of Alopecia Areata,” British Journal of Dermatology 155, no. 3 (2006): 515–521. 16911275 10.1111/j.1365-2133.2006.07377.x

[63] M. K. Tembhre and V. K. Sharma , “T‐Helper and Regulatory T‐Cell Cytokines in the Peripheral Blood of Patients With Active Alopecia Areata,” British Journal of Dermatology 169, no. 3 (2013): 543–548. 23607748 10.1111/bjd.12396

[64] E. Arca , U. Muşabak , A. Akar , A. H. Erbil , and H. B. Taştan , “Interferon‐Gamma in Alopecia Areata,” European Journal of Dermatology 14, no. 1 (2004): 33–36. 14965793

[65] E. Kasumagic‐Halilovic , A. Prohic , and J. Karamehic , “Serum Concentrations of Interferon‐Gamma (IFN‐g) in Patients With Alopecia Areata: Correlation With Clinical Type and Duration of the Disease,” Medicinski Arhiv 64, no. 4 (2010): 212–214. 21246917

[66] A. A. Alzolibani , Z. Rasheed , G. Bin Saif , M. S. Al‐Dhubaibi , and A. A. Al Robaee , “Altered Expression of Intracellular Toll‐Like Receptors in Peripheral Blood Mononuclear Cells from Patients With Alopecia Areata,” BBA Clinical 5 (2016): 134–142. 27114923 10.1016/j.bbacli.2016.03.006 PMC4826589

[67] M. Zöller , K. J. McElwee , M. Vitacolonna , and R. Hoffmann , “The Progressive State, in Contrast to the Stable or Regressive State of Alopecia Areata, is Reflected in Peripheral Blood Mononuclear Cells,” Experimental Dermatology 13, no. 7 (2004): 435–444. 15217364 10.1111/j.0906-6705.2004.00179.x

[68] N. Barahmani , A. Lopez , D. Babu , M. Hernandez , S. E. Donley , and M. Duvic , “Serum T Helper 1 Cytokine Levels are Greater in Patients With Alopecia Areata Regardless of Severity or Atopy,” Clinical and Experimental Dermatology 35, no. 4 (2010): 409–416. 19874320 10.1111/j.1365-2230.2009.03523.x

[69] S. I. Omar , A. M. Hamza , N. Eldabah , and D. A. Habiba , “IFN‐α and TNF‐α Serum Levels and Their Association With Disease Severity in Egyptian Children and Adults With Alopecia Areata,” International Journal of Dermatology 60, no. 11 (2021): 1397–1404. 34008204 10.1111/ijd.15658

[70] M. A. Atwa , N. Youssef , and N. M. Bayoumy , “T‐Helper 17 Cytokines (Interleukins 17, 21, 22, and 6, and Tumor Necrosis Factor‐α) in Patients With Alopecia Areata: Association With Clinical Type and Severity,” International Journal of Dermatology 55, no. 6 (2016): 666–672. 26235375 10.1111/ijd.12808

[71] A. Rossi , C. Cantisani , M. Carlesimo , et al., “Serum Concentrations of IL‐2, IL‐6, IL‐12 and TNF‐α in Patients With Alopecia Areata,” International Journal of Immunopathology and Pharmacology 25, no. 3 (2012): 781–788. 23058031 10.1177/039463201202500327

[72] D. J. Cua and C. M. Tato , “Innate IL‐17‐Producing Cells: The Sentinels of the Immune System,” Nature Reviews Immunology 10, no. 7 (2010): 479–489. 10.1038/nri2800 20559326

[73] D. B. O'Quinn , M. T. Palmer , Y. K. Lee , and C. T. Weaver , “Emergence of the Th17 Pathway and Its Role in Host Defense,” Advances in Immunology 99 (2008): 115–163. 19117534 10.1016/S0065-2776(08)00605-6

[74] A. Tanemura , N. Oiso , M. Nakano , S. Itoi , A. Kawada , and I. Katayama , “Alopecia Areata: Infiltration of Th17 Cells in the Dermis, Particularly around Hair Follicles,” Dermatology 226, no. 4 (2013): 333–336. 23838575 10.1159/000350933

[75] R. K. Gautam , Y. Singh , A. Gupta , P. Arora , A. Khurana , and A. Chitkara , “The Profile of Cytokines (IL‐2, IFN‐γ, IL‐4, IL‐10, IL‐17A, and IL‐23) in Active Alopecia Areata,” Journal of Cosmetic Dermatology 19, no. 1 (2020): 234–240. 31087753 10.1111/jocd.12970

[76] K. A. Bain , E. McDonald , F. Moffat , et al., “Alopecia Areata is Characterized by Dysregulation in Systemic Type 17 and Type 2 Cytokines, Which May Contribute to Disease‐Associated Psychological Morbidity,” British Journal of Dermatology 182, no. 1 (2020): 130–137. 30980732 10.1111/bjd.18008

[77] W. Liao , J. X. Lin , and W. J. Leonard , “IL‐2 Family Cytokines: New Insights Into the Complex Roles of IL‐2 as a Broad Regulator of T Helper Cell Differentiation,” Current Opinion in Immunology 23, no. 5 (2011): 598–604. 21889323 10.1016/j.coi.2011.08.003 PMC3405730

[78] P. Freyschmidt‐Paul , K. J. McElwee , R. Hoffmann , et al., “Reduced Expression of Interleukin‐2 Decreases the Frequency of Alopecia Areata Onset in C3H/HeJ Mice,” Journal of Investigative Dermatology 125, no. 5 (2005): 945–951. 16297194 10.1111/j.0022-202X.2005.23888.x

[79] Y. Teraki , K. Imanishi , and T. Shiohara , “Cytokines in Alopecia Areata: Contrasting Cytokine Profiles in Localized Form and Extensive Form (Alopecia Universalis),” Acta Dermato‐Venereologica 76, no. 6 (1996): 421–423. 8982401 10.2340/0001555576421423

[80] T. A. Waldmann , “Targeting the Interleukin‐15/Interleukin‐15 Receptor System in Inflammatory Autoimmune Diseases,” Arthritis Research & Therapy 6, no. 4 (2004): 174–177. 15225362 10.1186/ar1202 PMC464917

[81] P. Y. Perera , J. H. Lichy , T. A. Waldmann , and L. P. Perera , “The Role of Interleukin‐15 in Inflammation and Immune Responses to Infection: Implications for Its Therapeutic Use,” Microbes and Infection 14, no. 3 (2012): 247–261. 22064066 10.1016/j.micinf.2011.10.006 PMC3270128

[82] W. Ye , J. D. Young , and C. C. Liu , “Interleukin‐15 Induces the Expression of mRNAs of Cytolytic Mediators and Augments Cytotoxic Activities in Primary Murine Lymphocytes,” Cellular Immunology 174, no. 1 (1996): 54–62. 8929454 10.1006/cimm.1996.0293

[83] J. Fuentes‐Duculan , N. Gulati , K. M. Bonifacio , et al., “Biomarkers of Alopecia Areata Disease Activity and Response to Corticosteroid Treatment,” Experimental Dermatology 25, no. 4 (2016): 282–286. 26661294 10.1111/exd.12918

[84] M. A. El Aziz Ragab , E. M. Hassan , D. El Niely , and M. M. Mohamed , “Serum Level of Interleukin‐15 in Active Alopecia Areata Patients and Its Relation to Age, Sex, and Disease Severity,” Postepy Dermatol Alergol 37, no. 6 (2020): 904–908. 33603607 10.5114/ada.2020.102103 PMC7874869

[85] Y. Guo , W. Cao , and Y. Zhu , “Immunoregulatory Functions of the IL‐12 Family of Cytokines in Antiviral Systems,” Viruses. 11, no. 9 (2019): 772. 31443406 10.3390/v11090772 PMC6784021

[86] D. A. Vignali and V. K. Kuchroo , “IL‐12 Family Cytokines: Immunological Playmakers,” Nature Immunology 13, no. 8 (2012): 722–728. 22814351 10.1038/ni.2366 PMC4158817

[87] Y. Gong , Y. Zhao , X. Zhang , et al., “Serum Level of IL‐4 Predicts Response to Topical Immunotherapy With Diphenylcyclopropenone in Alopecia Areata,” Experimental Dermatology 29, no. 3 (2020): 231–238. 30047620 10.1111/exd.13758

[88] A. Waśkiel‐Burnat , M. Osińska , A. Salińska , et al., “The Role of Serum Th1, Th2, and Th17 Cytokines in Patients With Alopecia Areata: Clinical Implications,” Cells 10, no. 12 (2021). 10.3390/cells10123397 PMC8699846 34943905

[89] K. J. McElwee and R. Hoffmann , “Alopecia Areata—animal Models,” Clinical and Experimental Dermatology 27, no. 5 (2002): 410–417. 12190642 10.1046/j.1365-2230.2002.01075.x

[90] K. Katagiri , S. Arakawa , and Y. Hatano , “In Vivo Levels of IL‐4, IL‐10, TGF‐Beta1 and IFN‐Gamma mRNA of the Peripheral Blood Mononuclear Cells in Patients With Alopecia Areata in Comparison to Those in Patients With Atopic Dermatitis,” Archives of Dermatological Research 298, no. 8 (2007): 397–401. 17021766 10.1007/s00403-006-0700-2

[91] F. Siebenhaar , A. A. Sharov , E. M. Peters , et al., “Substance P as an Immunomodulatory Neuropeptide in a Mouse Model for Autoimmune Hair Loss (Alopecia Areata),” Journal of Investigative Dermatology 127, no. 6 (2007): 1489–1497. 17273166 10.1038/sj.jid.5700704

[92] T. Ito , N. Ito , A. Bettermann , Y. Tokura , M. Takigawa , and R. Paus , “Collapse and Restoration of MHC Class‐I‐Dependent Immune Privilege: Exploiting the Human Hair Follicle as a Model,” American Journal of Pathology 164, no. 2 (2004): 623–634. 14742267 10.1016/S0002-9440(10)63151-3 PMC1602279

[93] T. Breitkopf , B. K. Lo , G. Leung , et al., “Somatostatin Expression in Human Hair Follicles and Its Potential Role in Immune Privilege,” Journal of Investigative Dermatology 133, no. 7 (2013): 1722–1730. 23370538 10.1038/jid.2013.53

[94] R. Hoffmann , E. Wenzel , A. Huth , et al., “Cytokine mRNA Levels in Alopecia Areata Before and After Treatment With the Contact Allergen Diphenylcyclopropenone,” Journal of Investigative Dermatology 103, no. 4 (1994): 530–533. 7930677 10.1111/1523-1747.ep12395722

[95] D. Lee , S. K. Hong , S. W. Park , et al., “Serum Levels of IL‐18 and sIL‐2R in Patients With Alopecia Areata Receiving Combined Therapy With Oral Cyclosporine and Steroids,” Experimental Dermatology 19, no. 2 (2010): 145–147. 19758343 10.1111/j.1600-0625.2009.00937.x

[96] M. Shohat , D. Mimouni , D. Ben‐Amitai , et al., “In Vitro Cytokine Profile in Childhood Alopecia Areata and the Immunomodulatory Effects of AS‐101,” Clinical and Experimental Dermatology 30, no. 4 (2005): 432–434. 15953089 10.1111/j.1365-2230.2005.01817.x

[97] C. Bodemer , M. Peuchmaur , S. Fraitaig , L. Chatenoud , N. Brousse , and Y. De Prost , “Role of Cytotoxic T Cells in Chronic Alopecia Areata,” Journal of Investigative Dermatology 114, no. 1 (2000): 112–116. 10620125 10.1046/j.1523-1747.2000.00828.x

[98] T. Song , A. B. Pavel , H. C. Wen , et al., “An Integrated Model of Alopecia Areata Biomarkers Highlights both T(H)1 and T(H)2 Upregulation,” Journal of Allergy and Clinical Immunology 142, no. 5 (2018): 1631–1634. e13. 29981808 10.1016/j.jaci.2018.06.029

[99] R. P. Manimaran , S. Ramassamy , M. Rajappa , and L. Chandrashekar , “Therapeutic Outcome of Diphencyprone and Its Correlation With Serum Cytokine Profile in Alopecia Areata,” The Journal of Dermatological Treatment 33, no. 1 (2022): 324–328. 32249656 10.1080/09546634.2020.1752887

[100] O. Bilgic , A. Sivrikaya , A. Unlu , and H. C. Altinyazar , “Serum Cytokine and Chemokine Profiles in Patients With Alopecia Areata,” The Journal of Dermatological Treatment 27, no. 3 (2016): 260–263. 26367497 10.3109/09546634.2015.1093591

[101] C. Kim , J. M. Shin , D. Kim , et al., “Role of Substance P in Regulating Micro‐Milieu of Inflammation in Alopecia Areata,” Annals of Dermatology 34, no. 4 (2022): 270–277. 35948329 10.5021/ad.21.161 PMC9365655

[102] Ö. Aşkın , S. N. Yücesoy , E. Coşkun , B. Engin , and S. Serdaroğlu , “Evaluation of the Level of Serum Interleukins (IL‐2, IL‐4, IL‐15 andIL‐17) and Its Relationship With Disease Severity in Patients With Alopecia Areata,” Anais Brasileiros De Dermatologia 96, no. 5 (2021): 551–557. 34281739 10.1016/j.abd.2021.03.006 PMC8441470

[103] M. Kinori , M. Bertolini , W. Funk , et al., “Calcitonin Gene‐Related Peptide (CGRP) May Award Relative Protection from Interferon‐γ‐Induced Collapse of Human Hair Follicle Immune Privilege,” Experimental Dermatology 21, no. 3 (2012): 223–226. 22379970 10.1111/j.1600-0625.2011.01432.x

[104] R. Rossi , E. Del Bianco , D. Isolani , M. C. Baccari , and P. Cappugi , “Possible Involvement of Neuropeptidergic Sensory Nerves in Alopecia Areata,” Neuroreport 8, no. 5 (1997): 1135–1138. 9175100 10.1097/00001756-199703240-00015

[105] M. Bertolini , M. Pretzlaff , M. Sulk , et al., “Vasoactive Intestinal Peptide, Whose Receptor‐Mediated Signalling May be Defective in Alopecia Areata, ProvidesPprotection from Hair Follicle Immune Privilege Collapse,” British Journal of Dermatology 175, no. 3 (2016): 531–541. 27059672 10.1111/bjd.14645

[106] H. L. Yamaguchi , Y. Yamaguchi , and E. Peeva , “Pathogenesis of Alopecia Areata and Vitiligo: Commonalities and Differences,” International Journal of Molecular Sciences 25, no. 8 (2024): 4409. 38673994 10.3390/ijms25084409 PMC11049978

[107] T. Shimizu , Y. Mizue , R. Abe , H. Watanabe , and H. Shimizu , “Increased macrophage migration inhibitory factor (MIF) in the Sera of Patients With Extensive Alopecia Areata,” Journal of Investigative Dermatology 118, no. 3 (2002): 555–557. 11874501 10.1046/j.0022-202x.2001.01669.x

[108] M. M. Van Acker , R. R. Schwartz , K. Andrews , K. Seiffert‐Sinha , and A. A. Sinha , “Inheritance‐Specific Dysregulation of Th1‐ and Th17‐Associated Cytokines in Alopecia Areata,” Biomolecules 13, no. 9 (2023). 10.3390/biom13091285 PMC10527519 37759685

[109] C. N. Giordano and A. A. Sinha , “Cytokine Pathways and Interactions in Alopecia Areata,” European Journal of Dermatology 23, no. 3 (2013): 308–318. 23797621 10.1684/ejd.2013.2042

[110] M. Suárez‐Fariñas , B. Ungar , S. Noda , et al., “Alopecia Areata Profiling Shows TH1, TH2, and IL‐23 Cytokine Activation Without Parallel TH17/TH22 Skewing,” Journal of Allergy and Clinical Immunology 136, no. 5 (2015): 1277–1287. 26316095 10.1016/j.jaci.2015.06.032

[111] M. Fukuyama , T. Ito , and M. Ohyama , “Alopecia Areata: Current Understanding of the Pathophysiology and Update on Therapeutic Approaches, Featuring the Japanese Dermatological Association Guidelines,” Journal of Dermatology 49, no. 1 (2022): 19–36. 34709679 10.1111/1346-8138.16207

[112] Q. Gao , X. Liang , A. S. Shaikh , J. Zang , W. Xu , and Y. Zhang , “JAK/STAT Signal Transduction: Promising Attractive Targets for Immune, Inflammatory and Hematopoietic Diseases,” Current Drug Targets 19, no. 5 (2018): 487–500. 27928945 10.2174/1389450117666161207163054

[113] J. J. O'Shea and R. Plenge , “JAK and STAT Signaling Molecules in Immunoregulation and Immune‐Mediated Disease,” Immunity 36, no. 4 (2012): 542–550. 22520847 10.1016/j.immuni.2012.03.014 PMC3499974

[114] J. P. Sundberg , K. McElwee , M. A. Brehm , and L. Su , “Animal Models for Alopecia Areata: What and Where?,” Journal of Investigative Dermatology Symposium Proceedings 17, no. 2 (2015): 23–26. 26551940 10.1038/jidsymp.2015.35 PMC4722955

[115] C. H. Pratt , L. E. King Jr. , A. G. Messenger , A. M. Christiano , and J. P. Sundberg , “Alopecia Areata,” Nature reviews Disease primers 3 (2017): 17011. 10.1038/nrdp.2017.11 PMC5573125 28300084

[116] N. Ito , T. Ito , A. Kromminga , et al., “Human Hair Follicles Display a Functional Equivalent of the Hypothalamic‐Pituitary‐Adrenal Axis and Synthesize Cortisol,” Faseb Journal 19, no. 10 (2005): 1332–1334. 15946990 10.1096/fj.04-1968fje

[117] R. Paus , P. Arck , and S. Tiede , “(Neuro‐)Endocrinology of Epithelial Hair Follicle Stem Cells,” Molecular and Cellular Endocrinology 288, no. 1‐2 (2008): 38–51. 18423849 10.1016/j.mce.2008.02.023

[118] Y. Minokawa , Y. Sawada , and M. Nakamura , “Lifestyle Factors Involved in the Pathogenesis of Alopecia Areata,” International Journal of Molecular Sciences 23, no. 3 (2022): 1038. 35162962 10.3390/ijms23031038 PMC8835065

[119] Y. Sawada , N. Saito‐Sasaki , E. Mashima , and M. Nakamura , “Daily Lifestyle and Inflammatory Skin Diseases,” International Journal of Molecular Sciences 22, no. 10 (2021). 10.3390/ijms22105204 PMC8156947 34069063

[120] I. Khanimov , “Association between Smoking and Alopecia Areata: A Systematic Review and Meta‐Analysis,” International Journal of Dermatology 61, no. 1 (2022): e22–e24. 34468022 10.1111/ijd.15898

[121] C. W. Wang , M. Y. Wu , C. B. Chen , et al., “Clinical Characteristics and Immune Profiles of Patients With Immune‐Mediated Alopecia Associated With COVID‐19 Vaccinations,” Clinical Immunology 255 (2023): 109737. 37586672 10.1016/j.clim.2023.109737

[122] C. T. Richardson , M. S. Hayden , E. S. Gilmore , and B. Poligone , “Evaluation of the Relationship between Alopecia Areata and Viral Antigen Exposure,” American Journal of Clinical Dermatology 19, no. 1 (2018): 119–126. 28801732 10.1007/s40257-017-0312-y

[123] S. Chavez‐Alvarez , A. L. Villarreal‐Alfaro‐Lopez , O. Vazquez‐Martinez , and A. Villarreal‐Martinez , “Diffuse Alopecia Areata Associated With Weight‐Loss Pills,” International Journal of Trichology 11, no. 6 (2019): 236–237. 32030057 10.4103/ijt.ijt_101_19 PMC6984043

[124] J. M. Thompson , M. A. Mirza , M. K. Park , A. A. Qureshi , and E. Cho , “The Role of Micronutrients in Alopecia Areata: A Review,” American Journal of Clinical Dermatology 18, no. 5 (2017): 663–679. 28508256 10.1007/s40257-017-0285-x PMC5685931

[125] H. Park , C. W. Kim , S. S. Kim , and C. W. Park , “The Therapeutic Effect and the Changed Serum Zinc Level after Zinc Supplementation in Alopecia Areata Patients Who Had a Low Serum Zinc Level,” Annals of Dermatology 21, no. 2 (2009): 142–146. 20523772 10.5021/ad.2009.21.2.142 PMC2861201

[126] N. S. Abdel Fattah , M. M. Atef , and S. M. Al‐Qaradaghi , “Evaluation of Serum Zinc Level in Patients With Newly Diagnosed andRresistant Alopecia Areata,” International Journal of Dermatology 55, no. 1 (2016): 24–29. 26147750 10.1111/ijd.12769

[127] J. Kantor , L. J. Kessler , D. G. Brooks , and G. Cotsarelis , “Decreased Serum Ferritin is Associated With Alopecia in Women,” Journal of Investigative Dermatology 121, no. 5 (2003): 985–988. 14708596 10.1046/j.1523-1747.2003.12540.x

[128] W. Jin , H. Zheng , B. Shan , and Y. Wu , “Changes of Serum Trace Elements Level in Patients With Alopecia Areata: A Meta‐Analysis,” Journal of Dermatology 44, no. 5 (2017): 588–591. 28150385 10.1111/1346-8138.13705

[129] X. Lin , X. Meng , and Z. Song , “Vitamin D and Alopecia Areata: Possible Roles in Pathogenesis and Potential Implications for Therapy,” American journal of translational research 11, no. 9 (2019): 5285–5300. 31632510 PMC6789271

[130] C. Zhou , X. Li , C. Wang , and J. Zhang , “Alopecia Areata: An Update on Etiopathogenesis, Diagnosis, and Management,” Clinical Reviews in Allergy & Immunology 61, no. 3 (2021): 403–423. 34403083 10.1007/s12016-021-08883-0

[131] C. M. Hedrich and G. C. Tsokos , “Epigenetic Mechanisms in Systemic Lupus Erythematosus and Other Autoimmune Diseases,” Trends in Molecular Medicine 17, no. 12 (2011): 714–724. 21885342 10.1016/j.molmed.2011.07.005 PMC3225699

[132] Q. Deng , Y. Luo , C. Chang , H. Wu , Y. Ding , and R. Xiao , “The Emerging Epigenetic Role of CD8+T Cells in Autoimmune Diseases: A Systematic Review,” Frontiers in immunology 10 (2019): 856. 31057561 10.3389/fimmu.2019.00856 PMC6482221

[133] M. Zhao , G. Liang , X. Wu , et al., “Abnormal Epigenetic Modifications in Peripheral Blood Mononuclear Cells from Patients With Alopecia Areata,” British Journal of Dermatology 166, no. 2 (2012): 226–273. 10.1111/j.1365-2133.2011.10646.x 21936853

[134] E. H. C. Wang , G. M. DeStefano , A. V. Patel , et al., “Identification of Differentially Expressed miRNAs in Alopecia Areata that Target Immune‐Regulatory Pathways,” Genes and Immunity 18, no. 2 (2017): 100–104. 28300058 10.1038/gene.2017.4

[135] R. M. Trüeb and M. Dias , “Alopecia Areata: A Comprehensive Review of Pathogenesis and Management,” Clinical Reviews in Allergy & Immunology 54, no. 1 (2018): 68–87. 28717940 10.1007/s12016-017-8620-9

[136] T. A. Rodriguez and M. Duvic , “Onset of Alopecia Areata after Epstein‐Barr Virus Infectious Mononucleosis,” Journal of the American Academy of Dermatology 59, no. 1 (2008): 137–139. 18329131 10.1016/j.jaad.2008.02.005

[137] T. Y. Tu , R. Chang , J. N. Lai , et al., “Human Papillomavirus Symptomatic Infection Associated Fith Increased Risk of New‐Onset Alopecia Areata: A Nationwide Population‐Based Cohort Study,” Journal of Autoimmunity 119 (2021): 102618. 33714796 10.1016/j.jaut.2021.102618

[138] R. Paus and G. Cotsarelis , “The Biology of Hair Follicles,” New England Journal of Medicine 341, no. 7 (1999): 491–497. 10441606 10.1056/NEJM199908123410706

[139] S. Ji , Z. Zhu , X. Sun , and X. Fu , “Functional Hair Follicle Regeneration: An Updated Review,” Signal Transduction and Targeted Therapy 6, no. 1 (2021): 66. 33594043 10.1038/s41392-020-00441-y PMC7886855

[140] J. Z. Yenin , G. Serarslan , Z. Yönden , and K. T. Ulutaş , “Investigation of Oxidative Stress in Patients With Alopecia Areata and Its Relationship With Disease Severity, Duration, Recurrence and Pattern,” Clinical and Experimental Dermatology 40, no. 6 (2015): 617–621. 25524272 10.1111/ced.12556

[141] P. Öztürk , Ö. Arıcan , E. B. Kurutaş , and K. Mülayim , “Oxidative Stress Biomarkers and Adenosine Deaminase Over the Alopecic Area of the Patients With Alopecia Areata,” Balkan Medical Journal 33, no. 2 (2016): 188–192. 27403388 10.5152/balkanmedj.2016.16190 PMC4924963

[142] E. C. E. Wang , Z. Dai , A. W. Ferrante , C. G. Drake , and A. M. Christiano , “A Subset of TREM2(+) Dermal Macrophages Secretes Oncostatin M to Maintain Hair Follicle Stem Cell Quiescence and Inhibit Hair Growth,” Cell Stem Cell 24, no. 4 (2019): 654–669. e6. 30930146 10.1016/j.stem.2019.01.011

[143] D. Castellana , R. Paus , and M. Perez‐Moreno , “Macrophages Contribute to the Cyclic Activation of Adult Hair Follicle Stem Cells,” Plos Biology 12, no. 12 (2014): e1002002. 25536657 10.1371/journal.pbio.1002002 PMC4275176

[144] M. Maurer , E. Fischer , B. Handjiski , et al., “Activated Skin Mast Cells are Involved in Murine Hair Follicle Regression (Catagen),” Laboratory Investigation 77, no. 4 (1997): 319–332. 9354767

[145] T. Christoph , S. Müller‐Röver , H. Audring , et al., “The Human Hair Follicle Immune System: Cellular Composition and Immune Privilege,” British Journal of Dermatology 142, no. 5 (2000): 862–873. 10809841 10.1046/j.1365-2133.2000.03464.x

[146] R. Biran , A. Zlotogorski , and Y. Ramot , “The Genetics of Alopecia Areata: New Approaches, New Findings, New Treatments,” Journal of Dermatological Science 78, no. 1 (2015): 11–20. 25676427 10.1016/j.jdermsci.2015.01.004

[147] C. Jackow , N. Puffer , M. Hordinsky , J. Nelson , J. Tarrand , and M. Duvic , “Alopecia Areata and Cytomegalovirus Infection in Twins: Genes versus Environment?,” Journal of the American Academy of Dermatology 38, no. 3 (1998): 418–425. 9520023 10.1016/s0190-9622(98)70499-2

[148] T. A. Rodriguez , K. E. Fernandes , K. L. Dresser , and M. Duvic , “Concordance Rate of Alopecia Areata in Identical Twins Supports both Genetic and Environmental Factors,” Journal of the American Academy of Dermatology 62, no. 3 (2010): 525–527. 20159328 10.1016/j.jaad.2009.02.006

[149] R. C. Betz , L. Petukhova , S. Ripke , et al., “Genome‐Wide Meta‐Analysis in Alopecia Areata Resolves HLA Associations and Reveals Two New Susceptibility Loci,” Nature Communications 6 (2015): 5966. 10.1038/ncomms6966 PMC4451186 25608926

[150] L. Petukhova , M. Duvic , M. Hordinsky , et al., “Genome‐Wide Association Study in Alopecia Areata Implicates both Innate and Adaptive Immunity,” Nature 466, no. 7302 (2010): 113–117. 20596022 10.1038/nature09114 PMC2921172

[151] L. Petukhova and A. M. Christiano , “Functional Interpretation of Genome‐Wide Association Study Evidence in Alopecia Areata,” Journal of Investigative Dermatology 136, no. 1 (2016): 314–317. 26763452 10.1038/JID.2015.402 PMC4870380

[152] D. Jagielska , S. Redler , F. F. Brockschmidt , C. Herold , S. M. Pasternack , N. Garcia Bartels , et al., “Follow‐Up Study of the FirstGenome‐Wide Association Scan in Alopecia Areata: IL13 and KIAA0350 as Susceptibility Loci Supported With Genome‐Wide Significance,” Journal of Investigative Dermatology 132, no. 9 (2012): 2192–2197. 22534877 10.1038/jid.2012.129

[153] A. Oka , A. Takagi , E. Komiyama , et al., “Alopecia Areata Susceptibility Variant in MHC Region Impacts Expressions of Genes Contributing to Hair Keratinization and is Involved in Hair Loss,” EBioMedicine 57 (2020): 102810. 32580135 10.1016/j.ebiom.2020.102810 PMC7317227

[154] J. Fischer , F. Degenhardt , A. Hofmann , et al., “Genomewide Analysis of Copy Number Variants in Alopecia Areata in a Central European Cohort Reveals Association With MCHR2,” Experimental Dermatology 26, no. 6 (2017): 536–541. 27306922 10.1111/exd.13123

[155] L. Petukhova , R. M. Cabral , J. Mackay‐Wiggan , R. Clynes , and A. M. Christiano , “The Genetics of Alopecia Areata: What's New and How Will It Help Our Patients?,” Dermatologic Therapy 24, no. 3 (2011): 326–336. 21689242 10.1111/j.1529-8019.2011.01411.x

[156] A. M. Finner , “Alopecia Areata: Clinical Presentation, Diagnosis, and Unusual Cases,” Dermatologic Therapy 24, no. 3 (2011): 348–354. 21689244 10.1111/j.1529-8019.2011.01413.x

[157] H. Kimura , K. Nagase , and Y. Narisawa , “Perinevoid Alopecia: A Case Report and Literature Review,” British Journal of Dermatology 179, no. 4 (2018): 969–970. 29704462 10.1111/bjd.16709

[158] A. A. Navarini , S. Nobbe , and R. M. Trüeb , “Marie Antoinette Syndrome,” Archives of Dermatology 145, no. 6 (2009): 656. 19528420 10.1001/archdermatol.2009.51

[159] A. Rebora , “Alopecia Areata Incognita,” Journal of the American Academy of Dermatology 65, no. 6 (2011): 1228. 10.1016/j.jaad.2009.05.001 22082840

[160] A. Rebora , “Alopecia Areata Incognita: A Hypothesis,” Dermatologica 174, no. 5 (1987): 214–218. 2953632 10.1159/000249182

[161] A. Alessandrini , M. Starace , F. Bruni , et al., “Alopecia Areata Incognita and Diffuse Alopecia Areata: Clinical, Trichoscopic, Histopathological, and Therapeutic Features of a 5‐Year Study,” Dermatology Practical & Conceptual 9, no. 4 (2019): 272–277. 31723460 10.5826/dpc.0904a05 PMC6830548

[162] K. Chelidze and S. R. Lipner , “Nail Changes in Alopecia Areata: An Update and Review,” International Journal of Dermatology 57, no. 7 (2018): 776–783. 29318582 10.1111/ijd.13866

[163] Y. B. M. Roest , H. T. van Middendorp , A. W. M. Evers , P. C. M. van de Kerkhof , and M. C. Pasch , “Nail Involvement in Alopecia Areata: A Questionnaire‐Based Survey on Clinical Signs, Impact on Quality of Life and Review of the Literature,” Acta Dermato‐Venereologica 98, no. 2 (2018): 212–217. 28967977 10.2340/00015555-2810

[164] C. Pelzer and M. Iorizzo , “Alopecia Areata of the Nails: Diagnosis and Management,” Journal of Clinical Medicine 13, no. 11 (2024): 3292. 38893003 10.3390/jcm13113292 PMC11172645

[165] K. P. Huang , S. Mullangi , Y. Guo , and A. A. Qureshi , “Autoimmune, Atopic, and Mental Health Comorbid Conditions Associated With Alopecia Areata in the United States,” JAMA Dermatology 149, no. 7 (2013): 789–794. 23700152 10.1001/jamadermatol.2013.3049

[166] S. A. Muller and R. K. Winkelmann , “Alopecia Areata. An Evaluation of 736 Patients,” Archives of Dermatology 88 (1963): 290–297. 14043621 10.1001/archderm.1963.01590210048007

[167] A. Tosti , “Practice gaps. Alopecia Areata and Comorbid Conditions,” JAMA Dermatol 149, no. 7 (2013): 794. 23699633 10.1001/jamadermatol.2013.360

[168] H. Seyrafi , M. Akhiani , H. Abbasi , S. Mirpour , and A. Gholamrezanezhad , “Evaluation of the Profile of Alopecia Areata and the Prevalence of Thyroid Function Test Abnormalities and Serum Autoantibodies in Iranian Patients,” BMC Dermatology [Electronic Resource] 5 (2005): 11. 16259629 10.1186/1471-5945-5-11 PMC1280924

[169] T. Mubki , L. Rudnicka , M. Olszewska , and J. Shapiro , “Evaluation and Diagnosis of the Hair Loss Patient: Part II. Trichoscopic and Laboratory Evaluations,” Journal of the American Academy of Dermatology 71, no. 3 (2014): 431. e1‐e11. 10.1016/j.jaad.2014.05.008 25128119

[170] N. Barahmani , M. de Andrade , J. P. Slusser , et al., “Human Leukocyte Antigen Class II Alleles are Associated With Risk of Alopecia Areata,” Journal of Investigative Dermatology 128, no. 1 (2008): 240–243. 17637820 10.1038/sj.jid.5700973

[171] B. Zhou , M. Chen , S. Shang , and J. Zhao , “Association of CTLA‐4 Gene Polymorphisms and Alopecia Areata: A Systematic Review and Meta‐Analysis,” Biomarkers 27, no. 4 (2022): 338–348. 35254172 10.1080/1354750X.2022.2046855

[172] E. Olsen , M. Hordinsky , S. McDonald‐Hull , et al., “Alopecia Areata Investigational Assessment Guidelines. National Alopecia Areata Foundation,” Journal of the American Academy of Dermatology 40, no. 2 Pt 1 (1999): 242–246. 10025752 10.1016/s0190-9622(99)70195-7

[173] E. A. Olsen , M. K. Hordinsky , V. H. Price , et al., “Alopecia Areata Investigational Assessment Guidelines–part II. National Alopecia Areata Foundation,” Journal of the American Academy of Dermatology 51, no. 3 (2004): 440–447. 15337988 10.1016/j.jaad.2003.09.032

[174] C. G. Wambier and B. A. King , “Rethinking the Classification of Alopecia Areata,” Journal of the American Academy of Dermatology 80, no. 2 (2019): e45. 30244065 10.1016/j.jaad.2018.08.059

[175] B. A. King , N. A. Mesinkovska , B. Craiglow , et al., “Development of the Alopecia Areata Scale for Clinical Use: Results of an Academic‐Industry Collaborative Effort,” Journal of the American Academy of Dermatology 86, no. 2 (2022): 359–364. 34474079 10.1016/j.jaad.2021.08.043

[176] E. A. Olsen , J. Roberts , L. Sperling , et al., “Objective Outcome Measures: Collecting Meaningful Data on Alopecia Areata,” Journal of the American Academy of Dermatology 79, no. 3 (2018): 470–478. e3. 29128463 10.1016/j.jaad.2017.10.048 PMC7450487

[177] W. C. Cranwell , V. W. Lai , L. Photiou , et al., “Treatment of Alopecia Areata: An Australian Expert Consensus Statement,” Australasian Journal of Dermatology 60, no. 2 (2019): 163–170. 30411329 10.1111/ajd.12941

[178] D. Porter and J. L. Burton , “A Comparison of Intra‐Lesional Triamcinolone Hexacetonide and Triamcinolone Acetonide in Alopecia Areata,” British Journal of Dermatology 85, no. 3 (1971): 272–273. 5111692 10.1111/j.1365-2133.1971.tb07230.x

[179] A. Tosti , M. Iorizzo , G. L. Botta , and M. Milani , “Efficacy and Safety of a New lobetasol Propionate 0.05% Foam in Alopecia Areata: A Randomized, Double‐Blind Placebo‐Controlled Trial,” Journal of the European Academy of Dermatology and Venereology 20, no. 10 (2006): 1243–1247. 17062039 10.1111/j.1468-3083.2006.01781.x

[180] H. Zaher , H. I. Gawdat , R. A. Hegazy , and M. Hassan , “Bimatoprost versus Mometasone Furoate in the Treatment of Scalp Alopecia Areata: A Pilot Study,” Dermatology 230, no. 4 (2015): 308–313. 25765294 10.1159/000371416

[181] V. K. Sharma and S. Gupta , “Twice Weekly 5 Mg Dexamethasone Oral Pulse in the Treatment of Extensive Alopecia Areata,” Journal of Dermatology 26, no. 9 (1999): 562–565. 10535249 10.1111/j.1346-8138.1999.tb02049.x

[182] G. Açıkgöz , I. Ozmen , M. Cayırlı , Y. Yeniay , and O. Köse , “Pulse Methylprednisolone Therapy for the Treatment of Extensive Alopecia Areata,” The Journal of Dermatological Treatment 25, no. 2 (2014): 164–166. 23336179 10.3109/09546634.2013.768759

[183] M. Ait Ourhroui , B. Hassam , and I. Khoudri , “[Treatment of Alopecia Areata With Prednisone in a Once‐Monthly Oral Pulse],” Annales De Dermatologie Et De Venereologie 137, no. 8‐9 (2010): 514–518. 20804894 10.1016/j.annder.2010.06.002

[184] E. Altun , S. Yaylı , D. A. Arıca , L. B. Selcuk , and S. Bahadır , “Retrospective Analysis of Methylprednisolone Treatment Alone and in Combination With Methotrexate in Patients With Extensive Alopecia Areata,” Dermatologic Therapy 35, no. 10 (2022): e15776. 35986630 10.1111/dth.15776

[185] G. Mauduit , P. Lenvers , H. Barthélémy , and J. Thivolet , “[Treatment of Severe Alopecia Areata With Topical Applications of Cyclosporin A],” Annales De Dermatologie Et De Venereologie 114, no. 4 (1987): 507–510. 3619297

[186] P. Joly , “The Use of Methotrexate Alone or in Combination With Low Doses of Oral Corticosteroids in the Treatment of Alopecia Totalis or Universalis,” Journal of the American Academy of Dermatology 55, no. 4 (2006): 632–636. 17010743 10.1016/j.jaad.2005.09.010

[187] T. Much , “[Treatment of Alopecia Areata With Vitamin A Acid],” Zeitschrift Fur Hautkrankheiten 51, no. 23 (1976): 993–998. 1007363

[188] V. H. Price , “Double‐Blind, Placebo‐Controlled Evaluation of Topical Minoxidil in Extensive Alopecia Areata,” Journal of the American Academy of Dermatology 16, no. 3 Pt 2 (1987): 730–736. 3549809 10.1016/s0190-9622(87)70095-4

[189] V. C. Fiedler‐Weiss , “Topical Minoxidil Solution (1% and 5%) in the Treatment of Alopecia Areata,” Journal of the American Academy of Dermatology 16, no. 3 Pt 2 (1987): 745–748. 3549811 10.1016/s0190-9622(87)80003-8

[190] C. K. Rokhsar , J. L. Shupack , J. J. Vafai , and K. Washenik , “Efficacy of Topical Sensitizers in the Treatment of Alopecia Areata,” Journal of the American Academy of Dermatology 39, no. 5 Pt 1 (1998): 751–761. 9810892 10.1016/s0190-9622(98)70048-9

[191] A. Daunton and M. Harries , “Efficacy of Topical Dithranol (Dithrocream(®)) in the Treatment of Alopecia Areata: A Retrospective Case Series,” British Journal of Dermatology 180, no. 5 (2019): 1246–1247. 30536990 10.1111/bjd.17515

[192] S. C. Behrens‐Williams , U. Leiter , R. Schiener , M. Weidmann , R. U. Peter , and M. Kerscher , “The PUVA‐Turban as a New Option of Applying a Dilute Psoralen Solution Selectively to the Scalp of Patients With Alopecia Areata,” Journal of the American Academy of Dermatology 44, no. 2 (2001): 248–252. 11174382 10.1067/mjd.2001.110060

[193] G. Pagnanelli , A. Cavani , F. Canzona , and C. Mazzanti , “Mild Therapeutic Response of Alopecia Areata during Treatment of Psoriasis With Secukinumab,” European Journal of Dermatology 30, no. 5 (2020): 602–603. 33185532 10.1684/ejd.2020.3866

[194] E. Guttman‐Yassky , J. K. Nia , P. W. Hashim , et al., “Efficacy and Safety of Secukinumab Treatment in Adults With Extensive Alopecia Areata,” Archives of Dermatological Research 310, no. 8 (2018): 607–614. 30121698 10.1007/s00403-018-1853-5

[195] B. Yalici Armagan and N. Atakan , “New Onset Alopecia Areata During Secukinumab Therapy,” Dermatologic Therapy 32, no. 5 (2019): e13071. 31442356 10.1111/dth.13071

[196] L. R. Penzi , M. Yasuda , A. Manatis‐Lornell , D. Hagigeorges , and M. M. Senna , “Hair Regrowth in a Patient With Long‐Standing Alopecia Totalis and Atopic Dermatitis Treated With Dupilumab,” JAMA Dermatology 154, no. 11 (2018): 1358–1360. 30304403 10.1001/jamadermatol.2018.2976

[197] K. Harada , R. Irisawa , T. Ito , M. Uchiyama , and R. Tsuboi , “The Effectiveness of Dupilumab in Patients With alopecia Areata Who Have Atopic Dermatitis: A Case Series of Seven Patients,” British Journal of Dermatology 183, no. 2 (2020): 396–397. 32118289 10.1111/bjd.18976

[198] J. Chung , C. L. Slaught , and E. L. Simpson , “Alopecia Areata in 2 Patients Treated With Dupilumab: New Onset and Oorsening,” JAAD Case Reports 5, no. 8 (2019): 643–645. 31388527 10.1016/j.jdcr.2019.03.019 PMC6675971

[199] I. Salguero , M. Domingo , D. Suarez , and G. Roustan , “Dermatitis and Alopecia in a Patient Treated With Dupilumab: A New Adverse Effect?,” Clinical and Experimental Dermatology 44 (2018), e41–e43. 30536947 10.1111/ced.13858

[200] K. Flanagan , L. Sperling , and J. Lin , “Drug‐Induced Alopecia After Dupilumab Therapy,” JAAD Case Reports 5, no. 1 (2019): 54–56. 30560185 10.1016/j.jdcr.2018.10.010 PMC6289959

[201] E. Guttman‐Yassky , B. Ungar , S. Noda , et al., “Extensive Alopecia Areata is Reversed by IL‐12/IL‐23p40 Cytokine Antagonism,” Journal of Allergy and Clinical Immunology 137, no. 1 (2016): 301–304. 26607705 10.1016/j.jaci.2015.11.001

[202] A. Aleisa , Y. Lim , S. Gordon , et al., “Response to Ustekinumab in Three Pediatric Patients With Alopecia Areata,” Pediatric Dermatology 36, no. 1 (2019): e44–e45. 30338558 10.1111/pde.13699

[203] K. L. S. Kerkemeyer and R. Sinclair , “Treatment of Chronic Alopecia Areata With Tildrakizumab: An Open‐Label Pilot Study,” International Journal of Dermatology 59, no. 5 (2020): e136–e137. 32124974 10.1111/ijd.14826

[204] F. L. Duff , J. D. Bouaziz , E. Fontas , et al., “Low‐Dose IL‐2 for Treating Moderate to Severe Alopecia Areata: A 52‐Week Multicenter Prospective Placebo‐Controlled Study Assessing Its Impact on T Regulatory Cell and NK Cell Populations,” Journal of Investigative Dermatology 141, no. 4 (2021): 933–936. e6. 32941917 10.1016/j.jid.2020.08.015

[205] E. Castela , F. L.e Duff , C. Butori , et al., “Effects of Low‐Dose Recombinant Interleukin 2 to Promote T‐Regulatory Cells in Alopecia Areata,” JAMA Dermatology 150, no. 7 (2014): 748–751. 24872229 10.1001/jamadermatol.2014.504

[206] B. E. Strober , K. Siu , A. F. Alexis , et al., “Etanercept Does Not Effectively Treat Moderate to Severe Alopecia Areata: An Open‐Label Study,” Journal of the American Academy of Dermatology 52, no. 6 (2005): 1082–1084. 15928633 10.1016/j.jaad.2005.03.039

[207] L. Gorcey , E. A. Gordon Spratt , and M. C. Leger , “Alopecia Universalis Successfully Treated With Adalimumab,” JAMA Dermatology 150, no. 12 (2014): 1341–1344. 25322338 10.1001/jamadermatol.2014.1544

[208] H. M. Almohanna , A. A. Ahmed , J. W. Griggs , and A. Tosti , “Platelet‐Rich Plasma in the Treatment of Alopecia Areata: A Review,” Journal of Investigative Dermatology Symposium Proceedings 20, no. 1 (2020): S45–S49. 33099384 10.1016/j.jisp.2020.05.002

[209] W. Albalat and H. M. Ebrahim , “Evaluation of Platelet‐Rich Plasma vs Intralesional Steroid in Treatment of Alopecia Areata,” Journal of Cosmetic Dermatology 18, no. 5 (2019): 1456–1462. 31074201 10.1111/jocd.12858

[210] M. A. El Taieb , H. Ibrahim , E. A. Nada , and M. S. Al‐Din , “Platelets Rich Plasma versus Minoxidil 5% in Treatment of Alopecia areata: A Trichoscopic Evaluation,” Dermatologic Therapy 30, no. 1 (2017). 10.1111/dth.12437 27791311

[211] M. Croft , “Control of Immunity by the TNFR‐Related Molecule OX40 (CD134),” Annual Review of Immunology 28 (2010): 57–78. 10.1146/annurev-immunol-030409-101243 PMC2882161 20307208

[212] H. Iriki , H. Takahashi , and M. Amagai , “Diverse Role of OX40 on T Cells as a Therapeutic Target for Skin Diseases,” Journal of Investigative Dermatology 143, no. 4 (2023): 545–553. 36842860 10.1016/j.jid.2022.11.009

[213] Y. Fu , Q. Lin , Z. Zhang , and L. Zhang , “Therapeutic Strategies for the Costimulatory Molecule OX40 in T‐Cell‐Mediatedimmunity,” Acta Pharmaceutica Sinica B 10, no. 3 (2020): 414–433. 32140389 10.1016/j.apsb.2019.08.010 PMC7049610

[214] G. J. Webb , G. M. Hirschfield , and P. J. Lane , “OX40, OX40L and Autoimmunity: A Comprehensive Review,” Clinical Reviews in Allergy & Immunology 50, no. 3 (2016): 312–332. 26215166 10.1007/s12016-015-8498-3

[215] L. Y. Liu , B. G. Craiglow , F. Dai , and B. A. King , “Tofacitinib for the Treatment of Severe Alopecia Areata and Variants: A Study of 90 Patients,” Journal of the American Academy of Dermatology 76, no. 1 (2017): 22–28. 27816293 10.1016/j.jaad.2016.09.007

[216] A. K. Gupta , J. L. Carviel , and W. Abramovits , “Efficacy of Tofacitinib in Treatment of Alopecia Universalis in Two Patients,” Journal of the European Academy of Dermatology and Venereology 30, no. 8 (2016): 1373–1378. 27306107 10.1111/jdv.13598

[217] J. Mackay‐Wiggan , A. Jabbari , N. Nguyen , et al., “Oral Ruxolitinib Induces Hair Regrowth in Patients With Moderate‐To‐Severe Alopecia Areata,” JCI Insight 1, no. 15 (2016): e89790. 27699253 10.1172/jci.insight.89790 PMC5033756

[218] L. Pieri , P. Guglielmelli , and A. M. Vannucchi , “Ruxolitinib‐Induced Reversal of Alopecia Universalis in a Patient With Essential Thrombocythemia,” American Journal of Hematology 90, no. 1 (2015): 82–83. 25307179 10.1002/ajh.23871

[219] A. Jabbari , Z. Dai , L. Xing , et al., “Reversal of Alopecia Areata Following Treatment With the JAK1/2 Inhibitor Baricitinib,” EBioMedicine 2, no. 4 (2015): 351–355. 26137574 10.1016/j.ebiom.2015.02.015 PMC4486197

[220] L. Guo , S. Feng , B. Sun , X. Jiang , and Y. Liu , “Benefit and Risk Profile of Tofacitinib for the Treatment of Alopecia Areata: A Systemic Review and Meta‐Analysis,” Journal of the European Academy of Dermatology and Venereology 34, no. 1 (2020): 192–201. 31494993 10.1111/jdv.15937

[221] W. Damsky and B. A. King , “JAK Inhibitors in Dermatology: The Promise of a New Drug Class,” Journal of the American Academy of Dermatology 76, no. 4 (2017): 736–744. 28139263 10.1016/j.jaad.2016.12.005 PMC6035868

[222] B. King , J. Ko , S. Forman , et al., “Efficacy and Safety of the Oral Janus Kinase Inhibitor Baricitinib in the Treatment of Adults With Alopecia Areata: Phase 2 Results from a Randomized Controlled Study,” Journal of the American Academy of Dermatology 85, no. 4 (2021): 847–853. 34090959 10.1016/j.jaad.2021.05.050

[223] L. Y. Liu and B. A. King , “Ruxolitinib for the Treatment of Severe Alopecia Areata,” Journal of the American Academy of Dermatology 80, no. 2 (2019): 566–568. 30195572 10.1016/j.jaad.2018.08.040

[224] H. S. Park , M. W. Kim , J. S. Lee , et al., “Oral Tofacitinib Monotherapy in Korean Patients With Refractory Moderate‐To‐Severe Alopecia Areata: A Case Aeries,” Journal of the American Academy of Dermatology 77, no. 5 (2017): 978–980. 29029911 10.1016/j.jaad.2017.06.027

[225] K. Phan and D. F. Sebaratnam , “JAK Inhibitors for Alopecia Areata: A Systematic Review and Meta‐Analysis,” Journal of the European Academy of Dermatology and Venereology 33, no. 5 (2019): 850–856. 30762909 10.1111/jdv.15489

[226] Y. Renert‐Yuval and E. Guttman‐Yassky , “A Novel Therapeutic Paradigm for Patients With Extensive Alopecia Areata,” Expert Opinion on Biological Therapy 16, no. 8 (2016): 1005–1014. 27164008 10.1080/14712598.2016.1188076

[227] K. Bui , S. Polisetty , H. Gilchrist , S. M. Jackson , and J. Frederic , “Successful Treatment of Alopecia Universalis With Alefacept: A Case Report and Review of the Literature,” Cutis; Cutaneous Medicine for the Practitioner 81, no. 5 (2008): 431–434. 18543595

[228] B. E. Strober , K. Menon , A. McMichael , et al., “Alefacept for Severe Alopecia Areata: A Randomized, Double‐Blind, Placebo‐Controlled Study,” Archives of Dermatology 145, no. 11 (2009): 1262–1266. 19917955 10.1001/archdermatol.2009.264

[229] U. Kaelin , A. S. Hassan , L. R. Braathen , and N. Yawalkar , “Treatment of Alopecia Areata Partim Universalis With Efalizumab,” Journal of the American Academy of Dermatology 55, no. 3 (2006): 529–532. 16908369 10.1016/j.jaad.2006.05.062

[230] N. Taneja and S. Gupta , “Apremilast is Efficacious in Refractory Alopecia Areata,” The Journal of Dermatological Treatment 31, no. 7 (2020): 727–729. 31055978 10.1080/09546634.2019.1616046

[231] A. Keren , A. Shemer , Y. Ullmann , R. Paus , and A. Gilhar , “The PDE4 Inhibitor, Apremilast, Suppresses Experimentally Induced Alopecia Areata in Human Skin in Vivo,” Journal of Dermatological Science 77, no. 1 (2015): 74–76. 25530115 10.1016/j.jdermsci.2014.11.009

[232] P. Joly , A. Lafon , E. Houivet , et al., “Efficacy of Methotrexate Alone vs Methotrexate Plus Low‐Dose Prednisone in Patients With Alopecia Areata Totalis or Universalis: A 2‐Step Double‐Blind Randomized Clinical Trial,” JAMA Dermatology 159, no. 4 (2023): 403–410. 36884234 10.1001/jamadermatol.2022.6687 PMC9996454

[233] M. Ghassemi , N. Yazdanian , E. Behrangi , M. Jafari , and A. Goodarzi , “Comparison of Efficacy, Safety and Satisfaction of Latanoprost versus Minoxidil, Betamethasone and in Combination in Patients With Alopecia Areata: A Blinded Multiple Group Randomized Controlled Trial,” Dermatologic Therapy 35, no. 12 (2022): e15943. 36257912 10.1111/dth.15943

[234] R. K. Sivamani , D. Liepmann , and H. I. Maibach , “Microneedles and Transdermal Applications,” Expert Opinion on Drug Delivery 4, no. 1 (2007): 19–25. 17184159 10.1517/17425247.4.1.19

[235] J. C. Lee , M. A. Daniels , and M. Z. Roth , “Mesotherapy, Microneedling, and Chemical Peels,” Clinics in Plastic Surgery 43, no. 3 (2016): 583–595. 27363773 10.1016/j.cps.2016.03.004

[236] Y. A. Gomaa , M. J. Garland , F. J. McInnes , R. F. Donnelly , L. K. El‐Khordagui , and C. G. Wilson , “Microneedle/nanoencapsulation‐Mediated Transdermal Delivery: Mechanistic Insights,” European Journal of Pharmaceutics and Biopharmaceutics 86, no. 2 (2014): 145–155. 23461860 10.1016/j.ejpb.2013.01.026 PMC4074889

[237] C. H. Lee , Hair‐Growth Promoting Effect of Microneedle Roller Therapy, 2014.

[238] N.‐R. Kang , H.‐J. Yoon , and W.‐S. Ko , “Effects of Microneedle Therapy System (MTS) and Hwangryeonhaedoktang Pharmacopuncture Solution on Hair Growth in an Alopecia Model of C57BL/6N Mouse,” The Journal of Korean Medicine Ophthalmology and Otolaryngology and Dermatology 29, no. 1 (2016): 47–64.

[239] B. Chandrashekar , V. Yepuri , and V. Mysore , “Alopecia Areata‐Successful Outcome With Microneedling and Triamcinolone Acetonide,” Journal of Cutaneous and Aesthetic Surgery 7, no. 1 (2014): 63–64. 24761107 10.4103/0974-2077.129989 PMC3996798

[240] D. Pei , L. Chen , Y. Yao , L. Zeng , and G. Zhang , “Microneedling Combined With Compound Betamethasone in Treatment of Severe Alopecia Areata: A Case Report,” Frontiers in immunology 13 (2022): 939077. 35990624 10.3389/fimmu.2022.939077 PMC9381928

[241] R. M. Fertig , A. C. Gamret , J. Cervantes , and A. Tosti , “Microneedling for the Treatment of Hair Loss?,” Journal of the European Academy of Dermatology and Venereology 32, no. 4 (2018): 564–569. 29194786 10.1111/jdv.14722

[242] A. A. Farooqi , N. N. Desai , M. Z. Qureshi , et al., “Exosome Biogenesis, Bioactivities and Functions as New Delivery Systems of Natural Compounds,” Biotechnology Advances 36, no. 1 (2018): 328–334. 29248680 10.1016/j.biotechadv.2017.12.010

[243] R. Kalluri , “The Biology and Function of Exosomes in Cancer,” Journal of Clinical Investigation 126, no. 4 (2016): 1208–1215. 27035812 10.1172/JCI81135 PMC4811149

[244] B. Yang , Y. Chen , and J. Shi , “Exosome Biochemistry and Advanced Nanotechnology for Next‐Generation Theranostic Platforms,” Advanced Materials 31, no. 2 (2019): e1802896. 30126052 10.1002/adma.201802896

[245] D. M. Pegtel and S. J. Gould , “Exosomes,” Annual Review of Biochemistry 88 (2019): 487–514. 10.1146/annurev-biochem-013118-111902 31220978

[246] A. Joorabloo and T. Liu , “Engineering Exosome‐Based Biomimetic Nanovehicles for Wound Healing,” Journal of Controlled Release 356 (2023): 463–480. 36907562 10.1016/j.jconrel.2023.03.013

[247] R. Gowda , B. M. Robertson , S. Iyer , J. Barry , S. S. Dinavahi , and G. P. Robertson , “The Role of Exosomes in Metastasis and Progression of Melanoma,” Cancer Treatment Reviews 85 (2020): 101975. 32050108 10.1016/j.ctrv.2020.101975

[248] H. Yu , H. Feng , H. Zeng , et al., “Exosomes: The Emerging Mechanisms and Potential Clinical Applications in Dermatology,” International Journal of Biological Sciences 20, no. 5 (2024): 1778–1795. 38481799 10.7150/ijbs.92897 PMC10929203

[249] R. L. Rajendran , P. Gangadaran , S. S. Bak , et al., “Extracellular Vesicles Derived from MSCs Activates Dermal Papilla Cell in Vitro and Promotes Hair Follicle Conversion from Telogen to Anagen in Mice,” Scientific Reports 7, no. 1 (2017): 15560. 29138430 10.1038/s41598-017-15505-3 PMC5686117

[250] S. Hu , Z. Li , H. Lutz , et al., “Dermal Exosomes Containing miR‐218‐5p Promote Hair Regeneration by Regulating β‐Catenin Signaling,” Science Advances 6, no. 30 (2020): eaba1685. 32832660 10.1126/sciadv.aba1685 PMC7439409

[251] Y. Shi , J. Zhao , H. Li , et al., “A Drug‐Free, Hair Follicle Cycling Regulatable, Separable, Antibacterial Microneedle Patch for Hair Regeneration Therapy,” Advanced Healthcare Materials 11, no. 19 (2022): e2200908. 35817085 10.1002/adhm.202200908

[252] L. Y. Chou , K. Ming , and W. C. Chan , “Strategies for the Intracellular Delivery of Nanoparticles,” Chem. Soc. Rev. 40, no. 1 (2011): 233–245. 20886124 10.1039/c0cs00003e

[253] X. Li , X. Peng , M. Zoulikha , et al., “Multifunctional Nanoparticle‐Mediated Combining Therapy for Human Diseases,” Signal Transduction and Targeted Therapy 9, no. 1 (2024): 1. 38161204 10.1038/s41392-023-01668-1 PMC10758001

[254] R. Christmann , C. Thomas , N. Jager , et al., “Nanoparticle Targeting to Scalp Hair Follicles: New Perspectives for a Topical Therapy for Alopecia Areata,” Journal of Investigative Dermatology 140, no. 1 (2020): 243–246. e5. 31276676 10.1016/j.jid.2019.05.028

[255] B. Baroli , M. G. Ennas , F. Loffredo , M. Isola , R. Pinna , and M. A. López‐Quintela , “Penetration of Metallic Nanoparticles in Human Full‐Thickness Skin,” Journal of Investigative Dermatology 127, no. 7 (2007): 1701–1712. 17380118 10.1038/sj.jid.5700733

[256] W. Y. Jeong , S. Kim , S. Y. Lee , et al., “Transdermal Delivery of Minoxidil Using HA‐PLGA Nanoparticles for the Treatment in Alopecia,” Biomaterials Research 23 (2019): 16. 31695925 10.1186/s40824-019-0164-z PMC6824046

[257] M. Kuchukuntla , V. Palanivel , and A. Madhubabu , “Tofacitinib Citrate‐Loaded Nanoparticle Gel for the Treatment of Alopecia Areata: Response Surface Design, Formulation and In Vitro‐In Vivo Characterization,” Recent Advances in Drug Delivery and Formulation 17, no. 4 (2023): 314–331. 38031780 10.2174/0126673878264814231106094853

